# A high-throughput ChIP-Seq for large-scale chromatin studies

**DOI:** 10.15252/msb.20145776

**Published:** 2015-01-12

**Authors:** Christophe D Chabbert, Sophie H Adjalley, Bernd Klaus, Emilie S Fritsch, Ishaan Gupta, Vicent Pelechano, Lars M Steinmetz

**Affiliations:** 1 European Molecular Biology Laboratory, Genome Biology UnitHeidelberg, Germany; 2 Stanford Genome Technology CenterPalo Alto, CA, USA; 3 Department of Genetics, Stanford University School of MedicineStanford, CA, USA

**Keywords:** ChIP-Seq, chromatin, high-throughput, histone marks, histone methyltransferase

## Abstract

We present a modified approach of chromatin immuno-precipitation followed by sequencing (ChIP-Seq), which relies on the direct ligation of molecular barcodes to chromatin fragments, thereby permitting experimental scale-up. With Bar-ChIP now enabling the concurrent profiling of multiple DNA–protein interactions, we report the simultaneous generation of 90 ChIP-Seq datasets without any robotic instrumentation. We demonstrate that application of Bar-ChIP to a panel of *Saccharomyces cerevisiae* chromatin-associated mutants provides a rapid and accurate genome-wide overview of their chromatin status. Additionally, we validate the utility of this technology to derive novel biological insights by identifying a role for the Rpd3S complex in maintaining H3K14 hypo-acetylation in gene bodies. We also report an association between the presence of intragenic H3K4 tri-methylation and the emergence of cryptic transcription in a Set2 mutant. Finally, we uncover a crosstalk between H3K14 acetylation and H3K4 methylation in this mutant. These results show that Bar-ChIP enables biological discovery through rapid chromatin profiling at single-nucleosome resolution for various conditions and protein modifications at once.

## Introduction

While ChIP-Seq and ChIP-on-chip remain the standard methods for global detection of binding sites associated with protein factors and histone chemical modifications, these approaches only allow profiling of a single protein modification per experiment. Given the vast number of post-translational modifications (PTM) implicated in biological processes (Kouzarides, 2007; Li *et al*, 2007a; Misteli & Soutoglou, 2009; Ransom *et al*, 2010; MacAlpine & Almouzni, 2013), their potential combinations on multiple amino acid residues and the possibility of interactions between these modifications, investigating chromatin biology requires generating ChIP-Seq or ChIP-on-chip data for numerous marks and across multiple physiological conditions. Therefore, studies of histone PTM dynamics necessitate a considerable number of individual experiments. For instance, a recent study, reporting the previously underestimated role of histone PTM in yeast stress response, required a total of thirty ChIP-on-chip assays to profile five histone marks across multiple points of a time course (Weiner *et al*, 2012). Similarly, determining histone modification patterns associated with cellular states in human cells often requires several hundred ChIP-Seq experiments (ENCODE Project Consortium, [Bibr b202]). High-throughput ChIP-Seq approaches based on the use of robotic tools have been developed (Garber *et al*, 2012; Aldridge *et al*, 2013) but remain restricted to a discrete number of laboratories that have access to the required instrumentation. Therefore, generation of comparative data from large-scale, genome-wide ChIP-Seq experiments remains cumbersome and costly to most laboratories.

Here, we propose to implement a DNA barcoding step prior to chromatin immuno-precipitation to increase the speed and performance of ChIP-Seq experiments. The concept of DNA barcoding was recently applied to chromatin biology to investigate the biochemical mechanisms underlying the activity of histone PTM enzymes albeit in an *in vitro* context (Nguyen *et al*, 2014). Our barcoded high-throughput ChIP-Seq (Bar-ChIP) method relies on the direct barcoding of mono-nucleosomes derived from the isolation of yeast cell chromatin. As a proof of concept, we applied Bar-ChIP to the study of histone PTM in several *Saccharomyces cerevisiae* chromatin modifier mutants, which were profiled in parallel for five distinct histone modifications, thereby compressing 90 individual ChIP-Seq experiments into five. We show that barcoding and multiplexing of chromatin samples prior to immuno-precipitation greatly reduces the workload needed to perform ChIP-Seq experiments and permits a direct comparison between biological samples that are interrogated for specific histone PTM.

We demonstrate that this method enables the faithful capture of histone PTM in *S. cerevisiae* in a genome-wide manner and confirm that the histone deacetylase complex, Rpd3S, upon activation by the methyl-transferase Set2, maintains a low level of histone acetylation in gene bodies. We show that Rpd3S activity is not restricted to histone H4 and lysine 9 and 56 of H3 (Venkatesh *et al*, 2012), but also targets H3K14. In addition, we report for the first time differential methylation on lysine 4 of H3 in a *set2Δ* mutant and demonstrate that intragenic H3K4me3 associates with the emergence of cryptic transcription. Validation of the newly discovered histone PTM trends underscores the power of Bar-ChIP to quickly and accurately screen for histone PTM patterns in multiple biological samples at once, which would not be feasible with traditional ChIP-Seq approaches.

## Results

### Bar-ChIP captures genome-wide distribution of H3K4me3

**Figure 1:**
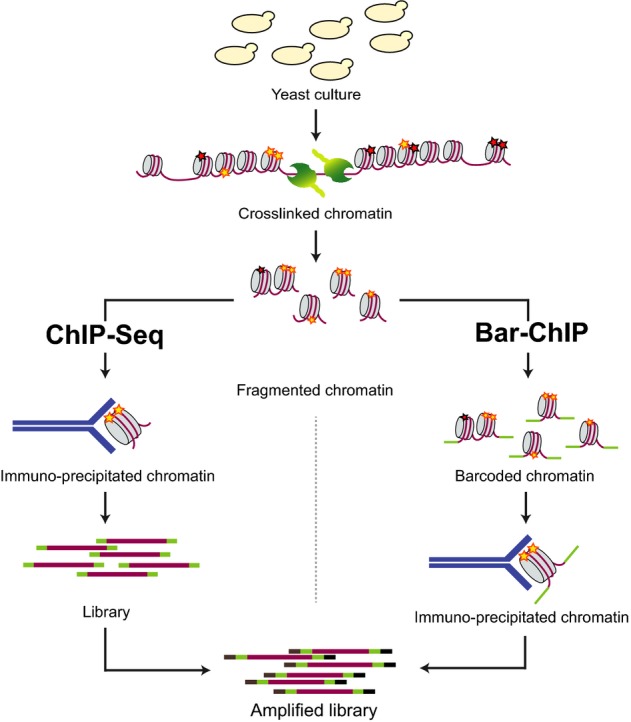
Comparative representation of the ChIP-Seq and Bar-ChIP workflows Yeast cultures are crosslinked using formaldehyde. Chromatin is then extracted and fragmented using micrococcal nuclease (MNase) digestion. In a classical ChIP-Seq protocol, MNase-treated chromatin is directly immuno-precipitated with an antibody against the protein modification or factor of interest. Recovered DNA is then barcoded and used to generate an amplified DNA library ready for paired-end sequencing. In the Bar-ChIP protocol, fragmented chromatin is barcoded through ligation of molecular barcodes prior to immuno-precipitation. DNA recovered from the IP can directly be amplified by PCR using Illumina primers and deep-sequenced. The presence of the barcoding step early in the workflow allows for multiplexing of IP assays.

Bar-ChIP relies on the direct ligation of DNA molecular barcodes to fragmented chromatin using an adapted version of the classical Illumina DNA library preparation protocol (Fig1). Barcoding of fragmented chromatin occurs prior to the immuno-precipitation step, hereby allowing for the pooling of numerous independently barcoded chromatin samples that will then be simultaneously subjected to the same assay. Therefore, for every protein or modification of interest, a single immuno-precipitation may be possible independently of the number of examined biological samples. In the current study, micrococcal nuclease (MNAse) was used to digest yeast crosslinked chromatin and isolate mono-nucleosome fractions to address the genome-wide distribution of histone post-translational modifications (PTM).

To validate our approach, we first evaluated the impact of chromatin barcoding on the recovery of genome-wide patterns of H3K4me3. This mark was selected because of its prominent and well-characterized distribution on promoter regions (Shilatifard, 2008) and its association with active transcription (Pokholok *et al*, 2005; Hon *et al*, 2009). H3K4me3 was profiled for three independent biological replicates of *S. cerevisiae* cultures grown in rich media (YPD) using both Bar-ChIP and classical ChIP-Seq methods. Both barcoded and non-barcoded fractions, containing mono-, di- and tri-nucleosomes, were then subjected to immuno-precipitation with an antibody specific for the histone mark (Fig1).

IP-DNA libraries derived from both protocols were PCR-amplified and deep-sequenced on an Illumina HiSeq 2000 instrument using paired-end technology. Input libraries were systematically included to control for potential biases in local chromatin solubility, enzyme accessibility and/or PCR amplification. No size-specific selection of mono-nucleosomal fragments was performed (Henikoff *et al*, 2011); however, only pairs of unambiguously mapped reads stemming from mono-nucleosomal particles were considered for downstream analysis (Supplementary Fig. S1). Interestingly, mono-nucleosomes deriving from the fragments generated following the Bar-ChIP protocol and recovered after the IP were slightly longer (Supplementary Fig. S1).

**Figure 2:**
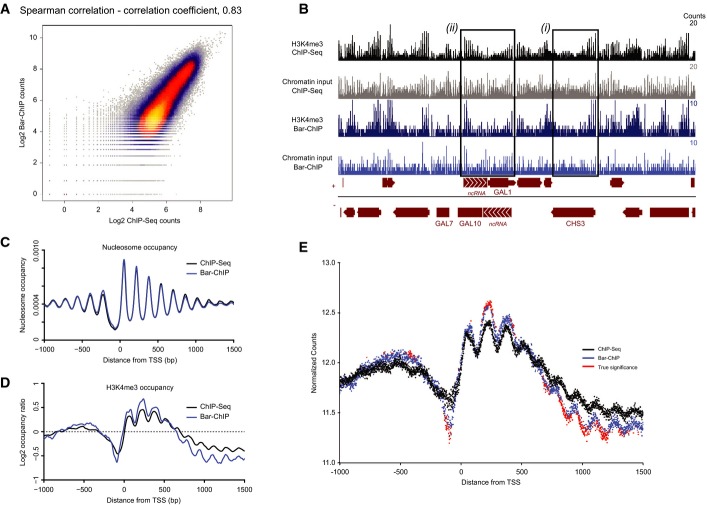
Direct comparison of the Bar-ChIP and ChIP-Seq methods

A very good reproducibility was observed between biological replicates with a mean Spearman's correlation coefficient of 0.88 ± 0.04 for both ChIP- and Bar-ChIP-Seq (Supplementary Fig. S2). Additionally, a high correlation between the two techniques was obtained for each IP DNA and input DNA, with a mean Spearman's correlation coefficient of 0.79 ± 0.07 (Fig2A; Supplementary Fig. S3).

Scatterplot illustrating the correlation between reads counts in the Bar-ChIP and ChIP-Seq datasets obtained for the same biological sample. 100 bp bins were used to compute these counts.

Snapshot of coverage tracks of H3K4me3 around the *GAL10* locus obtained using ChIP-Seq and Bar-ChIP protocols. Tracks corresponding to the associated chromatin inputs are also displayed. For a highly expressed gene such as *CHS3* (black box (i)), H3K4me3 signal is much stronger at the 5′ end of the gene as compared to the input signal, which spreads along the entire gene body. On the other hand, while input signals display a rather homogeneous nucleosome spread on the *GAL1* and *GAL10* genes, H3K4me3 immuno-precipitation clearly shows an enrichment of the mark to the 3′ end of the gene (black box (ii)). This corresponds to the presence of actively transcribed antisense non-coding RNAs over the *GAL10* and *GAL1* genes that are responsible for the inhibition of GAL expression in dextrose-rich medium. Bedgraph format displaying the number of counts per base pair.

TSS plot representing nucleosome occupancy around the TSS of annotated genes as observed in the chromatin input of both ChIP-Seq and Bar-ChIP methods. Occupancy levels are plotted as a function of the distance to the TSS. Midpoint of a nucleosome was approximated as being the center of the genomic locus intercepted by a read pair. Nucleosome counts were determined at each position around annotated TSS and estimated across all genes. Resulting counts were divided by the total number of observed nucleosomes to provide the genome-wide distribution of nucleosomes and occupancy at each position around the TSS.

TSS plot representing H3K4me3 occupancy (normalized by chromatin input) around the TSS of annotated genes as observed in the ChIP-Seq and Bar-ChIP datasets.

Evaluation of H3K4me3 counts with statistically significant difference between ChIP-Seq and Bar-ChIP. The mean normalized counts across all biological replicates are plotted as a function of the distance to the annotated TSS. Differential counts with statistical significance are indicated in red. Significant differences were called with the DESeq2 package after computing local FDR values based on the DESeq2 *P*-values. Only the positions with a log2 fold change greater than 0.05 and a local FDR smaller than 0.2 were considered significant.

Signals for the presence of the H3K4me3 mark were equally well recovered by the two methods as confirmed by the high correlation obtained for regions with the PTM enrichment (Supplementary Fig. S4) and by manual inspection of the coverage tracks for selected loci. For instance, the *CHS3* promoter is enriched in H3K4me3 in comparison to total nucleosome occupancy (Fig2B). Similarly, the *GAL10* locus harbored high H3K4me3 levels on its 3′ end, consistent with active transcription of an antisense non-coding RNA that acts in *GAL10* repression during growth in dextrose-containing medium (Houseley *et al*, 2008) (Fig2B). These observations underline the comparable acquisition of biological information with either technique.

To compare the two ChIP approaches at a genome-wide level, the distributions of nucleosomes derived from both classical and Bar-ChIP were analyzed within −1,000 bp to + 1,500 bp around annotated transcription start sites (TSS) (Fig2C). The map of nucleosome occupancy for nucleosomal DNA produced using classical ChIP-Seq revealed an array of 150 bp-spaced nucleosomes on either side of the TSS, as previously reported (Yuan, 2005; Jiang & Pugh, 2009; Weiner *et al*, 2010). An identical trend of nucleosome occupancy was observed for nucleosomal DNA generated using Bar-ChIP, confirming our ability to capture nucleosome distributions even when DNA adapters are directly ligated to fragmented chromatin (Fig2C).

Mapping of H3K4me3 occupancy around annotated TSS for both protocols showed a clear enrichment for nucleosomes carrying the PTM at the 5′ end of genes (up to + 500 bp) (Fig2D). Local statistical differences between the distributions of the histone mark obtained by either traditional ChIP or Bar-ChIP were computed using the DESeq2 package (Love *et al*, 2014) to provide an estimate of potential disparities between the two methods (see Materials and Methods). We found that enrichment signals obtained with Bar-ChIP were of slightly greater amplitude than those generated with classical ChIP-Seq (Fig2D and E; Supplementary Figs S5 and S6). This suggests that regions enriched in H3K4me3-marked nucleosomes were more prominently detected with Bar-ChIP, which tended to also exaggerate the depletion signals originating from H3K4me3-poor regions. Despite these differences, the same genomic regions were identified by both techniques for H3K4me3 enrichment or depletion, indicating that Bar-ChIP faithfully captured the distribution of H3K4me3-marked nucleosomes.

### Bar-ChIP enables rapid and simultaneous generation of ChIP-Seq data sets

**Figure 3:**
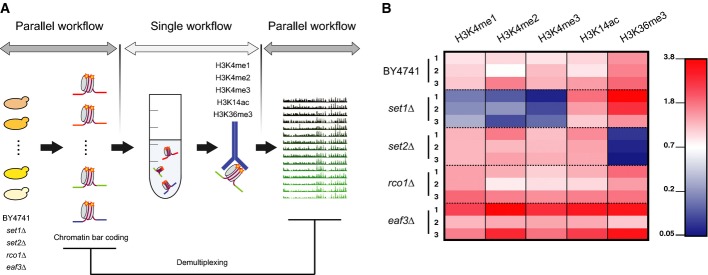
Highly multiplexed ChIP experiment based on the Bar-ChIP protocol

Current protocols for ChIP-Seq suffer from several practical constraints, including cost, laboriousness, as well as the limitation of having only one modification or protein profiled per assay. To demonstrate the multiplexing potential of Bar-ChIP and its suitability for rapid and systematic profiling of histone modifications across multiple yeast samples, the approach was applied to five histone PTM in a panel of four *S. cerevisiae* chromatin modifier mutants using biological triplicates (Fig3A). As these mutants and histone marks have been mostly characterized using ChIP-on-chip methods, our objective was to comparatively assess the resolution provided by Bar-ChIP and address the interplay between histone modifications.

Schematic representation of the experimental design. Cultures corresponding to distinct yeast strains were harvested, crosslinked and their MNase-treated chromatin was barcoded to enable sample tracking. Aliquots from each of the barcoded chromatin samples were pooled together prior to immuno-precipitation against the histone modifications of interest. DNA recovered from each IP was amplified and sequenced using paired-end technology. Finally, barcode sequences were used to demultiplex sequencing datasets and attribute each read to the proper biological sample.

Normalized proportion of reads attributed to each strain. One sequencing lane corresponded to multiplexed samples submitted to one IP assay. For each sequencing lane, read counts attributed to each biological sample were first divided by the total number of reads recovered from the lane (Supplementary Fig. S6). For each biological sample, the resulting ratio was normalized using the proportion of reads in the chromatin input lane that was attributed to that biological sample to correct for biases in the initial pooling of fragmented chromatin samples. Note that each *set1Δ* library represents less than 0.17% of the total number of reads recovered for the IPs against H3K4 methylation while the *set2Δ* libraries represent not more than 0.8% of the total reads recovered for the IP against H3K36me3. Absolute numbers for recovered sequencing reads are indicated in Supplementary Fig. S7.

To ensure consistency in the data, all these chromatin-associated mutants were derived from the BY4741 strain background, from which the initial *Saccharomyces cerevisiae* deletion collection was generated (Winzeler, 1999). Our set of mutants included *set1*Δ, deleted for Set1p, a component of the COMPASS complex, which contains a SET domain and is the only protein capable of catalyzing the deposition of mono-, di- and trimethyl groups on lysine 4 of H3 in *S. cerevisiae* (Roguev *et al*, 2001; Krogan *et al*, 2002; Santos-Rosa *et al*, 2002). Set1p is strongly active at the 5′ end of actively transcribed genes, where it results in peaks of H3K4me3. Loss of Set1p results in the absence of methylation on H3K4 and in the local emergence of new transcripts from previously silent loci (Venkatasubrahmanyam *et al*, 2007; Lenstra *et al*, 2011).

Another SET mutant analyzed in our study was *set2*Δ, as Set2p is the only histone methyltransferase responsible for deposition of methyl groups on lysine 36 of H3 (H3K36me1, 2, 3) in *S. cerevisiae* (Strahl *et al*, 2002). Set2p associates with the elongating form of RNA polymerase II, when it is phosphorylated on serine 2 of its carboxyl-terminal domain (CTD) (Krogan *et al*, 2003; Li, 2003). Set2p then deposits the elongation mark H3K36me3 on nucleosomes toward the 3′ end of genes (Pokholok *et al*, 2005). Deposition of this mark enables activation of the deacetylase complex, Rpd3S, which maintains low histone acetylation levels within the coding region of transcribed genes, thereby preventing cryptic transcript initiation (Carrozza *et al*, 2005; Keogh *et al*, 2005; Li *et al*, 2007c; Drouin *et al*, 2010). H3K36me3-dependent regulation of Rpd3S involves two subunits: Rco1p, which possesses a PHD zinc finger domain, permitting binding to histones regardless of their PTM, and a chromodomain-containing protein, Eaf3p, that recognizes the H3K36me3 mark (Li *et al*, 2007b). To better understand the interactions between these proteins, the interplay between their enzymatic activities and the presence of specific histone marks, both *rco1*Δ and *eaf3*Δ mutants, were profiled for histone PTM.

Given the functions of the proteins described above, five distinct histone modifications were selected: H3K14ac and H3K4me3, two marks located at the 5′ end of genes and associated with active transcription; H3K36me3, associated with transcription elongation; and H3K4me2 and H3K4me1 marks, whose roles in transcriptional processes are not as well delineated. Due to the complete absence of methylation of H3K4 in *set1*Δ, this mutant was used as an internal control for the IP specificity when profiling H3K4me1, H3K4me2 and H3K4me3. Similarly, the *set2*Δ mutant constituted a control for the IP specificity of H3K36me3.

For each yeast strain, three biological replicates were grown in YPD, crosslinked, and their chromatin was subjected to MNase-mediated fragmentation. Chromatin samples were then barcoded and pooled prior to parallel immuno-precipitation. Recovered DNA libraries were PCR-amplified and deep-sequenced using paired-end technology, with one library per sequencing lane. Each amplified library corresponded to DNA products obtained from one IP against a specific histone mark, albeit for 15 biological samples at once. As before, input DNAs were also sequenced to control for potential biases in MNase accessibility and sequencing. Consequently, the Bar-ChIP method applied to a unique experiment yielded the equivalent of 90 ChIP-Seq datasets with only five chromatin IP assays (Fig3A).

One hundred and twenty million reads were recovered on average from each sequencing lane and demultiplexed. The distribution of reads between the 15 biological samples reliably reflected the original chromatin composition expected for each pool from the distinct ChIP assays (Supplementary Fig. S7). Of 4 to 9 million unique molecules were retrieved per pool for each histone modification, indicative of a rather low resolution of the data (Supplementary Fig. S8). However, normalization of the data using the input read counts for every strain showed a clear depletion in H3K4me1, 2, 3 and H3K36me3 levels for the *set1*Δ and *set2*Δ strains, respectively, as was expected (Fig3B). These results confirmed our capacity to perform chromatin IP of barcoded and pooled chromatin fragments, suggesting that Bar-ChIP can be used to study the genome-wide patterns of histone marks.

### Multiplexed experiments provide overview of genome-wide distribution of histone marks

To evaluate the value of Bar-ChIP for investigating chromatin-associated processes, the 90 datasets were explored for potential interactions between histone PTM. For each profiled strain, data from the biological replicates were pooled together, thereby increasing resolution and permitting an accurate comparison between datasets.

**Figure 4:**
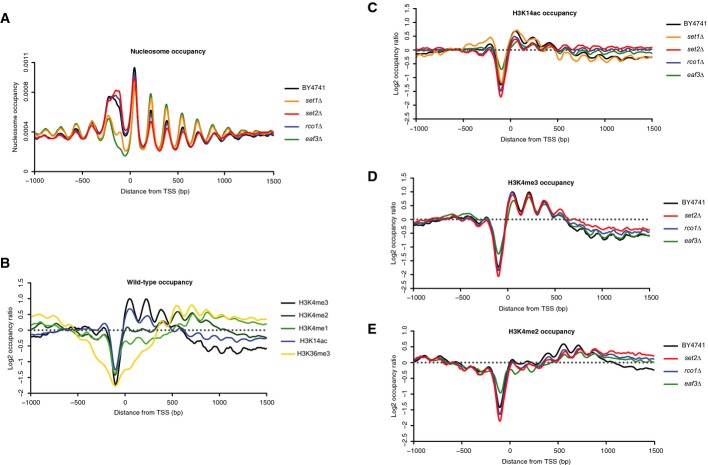
TSS plots depicting the distribution of the histone post-translational modifications profiled in the multiplexing experiment

Analysis of the genome-wide nucleosome distributions for wild-type and mutant strains showed that *eaf3*Δ and *set1*Δ chromatin was generally more sensitive to MNase digestion (Fig4A), as suggested by the disappearance of the di-nucleosome and widening of the mono-nucleosome signal reproducibly observed by bioanalyzer (Supplementary Fig. S9). Maps of nucleosome occupancy revealed that nucleosome-depleted regions located approximately 100 bp upstream of annotated TSS were more pronounced and 50 to 100 bp wider in *eaf3*Δ and *set1*Δ. In contrast, each other mutant exhibited typical nucleosome organization around the TSS (Fig4A; Supplementary Fig. S10), except for an unusually wide profile of the −1 nucleosome. We attribute this difference to a greater heterogeneity of the fragments obtained by MNase digestion of the corresponding regions. Additionally, the average size of mono-nucleosome fragments was smaller in the two mutants despite simultaneous treatment of all samples with the same amount of MNase (Supplementary Fig. S11). This difference was taken into consideration when performing the comparative downstream analyses.

Nucleosome occupancy around annotated TSS for the wild-type and mutant yeast strains profiled in the study.

Occupancy of the various marks profiled in the experiment in the wild-type strain BY4741.

H3K14ac (C), H3K4me3 (D) and H3K4me2 (E) occupancies around the annotated TSS in the mutants profiled in the study. Data information: Histone mark occupancies were normalized using the counts corresponding to chromatin inputs.

These maps of nucleosome occupancy were then used to examine enrichment profiles around the TSS obtained for the various histone PTM and yeast strains. Enrichments obtained in the wild-type strain for each profiled histone mark confirmed the specificity and consistency of the IPs (Fig4B). As expected, H3K14ac and H3K4me3 peaked at the 5′ end of genes. High levels of H3K4me2 were located about 500 bp after annotated transcription initiation sites, as previously reported in single gene and genome-wide studies (Santos-Rosa *et al*, 2002; Ng *et al*, 2003; Liu *et al*, 2005; Pokholok *et al*, 2005). H3K4me1-enriched nucleosomes were present ∽600 bp downstream of the TSS. Finally, H3K36me3, the mark associated with elongating RNA polymerase II, was enriched near the 3′ end of genes (Pokholok *et al*, 2005; Li *et al*, 2007a). Despite the aforementioned wider profile of the −1 nucleosome, comparison of the H3K4me3 enrichment patterns obtained in both comparative and multiplex experiments did not show any significant difference (Supplementary Fig. S12).

Additionally, to assess the possibility of cross-contamination during sample pooling, the enrichment patterns derived from the remnant reads for H3K4 methylation and H3K36me3 in *set1*Δ and *set2*Δ, respectively, were examined. These generally did not resemble those of the wild-type strain or of the other mutants (Supplementary Fig. S13), although traces of H3K4 methylation were still detected in *set1*Δ (Supplementary Fig. S13E and F), albeit corresponding to a very low number of sequencing reads (Supplementary Fig. S7). Altogether, these observations confirmed that IP experiments performed on barcoded chromatin were successful in capturing an enriched fraction of nucleosomes carrying the targeted histone PTM.

The profiles of H3K36me3 were similar between wild-type and *set1*Δ, *eaf3*Δ and *rco1*Δ strains, except for an exaggerated depletion around the TSS for *set1*Δ (Supplementary Fig. S14). The pattern of H3K14 acetylation in this mutant closely resembled that of the wild-type strain. In contrast, *set2*Δ, *eaf3*Δ and *rco1*Δ mutants displayed an equal and comparable distribution of the acetylation mark along the entire gene body, reflecting the globally high levels of histone acetylation present genome-wide upon deletion of these chromatin-associated genes (Fig4C).

In the *set2*Δ mutant, distributions of H3K4me3, H3K4me2 and H3K4me1 to a lesser extent differed from those observed in the wild-type strain (Fig4D and E; Supplementary Fig. S14), suggesting that the deletion of *SET2* impacts the methylation profile of H3K4. While the main peak of H3K4me3 occupancy was present near the 5′ end of genes, similar to the wild-type profile, a modest enrichment of H3K4 trimethylation was detected at the 3′ end of genes, beyond the first 500 bp. This was also confirmed by manual examination of gene coverage tracks (Supplementary Fig. S15). The peak for H3K4me2 was slightly shifted toward the 3′ end of genes in comparison to the wild-type strain, such that high levels of the mark were then maintained in the 3′ region of gene bodies beyond 1,000 bp, while regions between 500 bp and 1,000 bp appeared to be depleted in H3K4 di-methylation. Interestingly, this trend of H3K4me2 was conserved in the *eaf3*Δ and *rco1*Δ mutants, albeit moderately.

### Altered distribution of chromatin marks in the *set2*Δ mutant associates with the emergence of cryptic transcription

**Figure 5:**
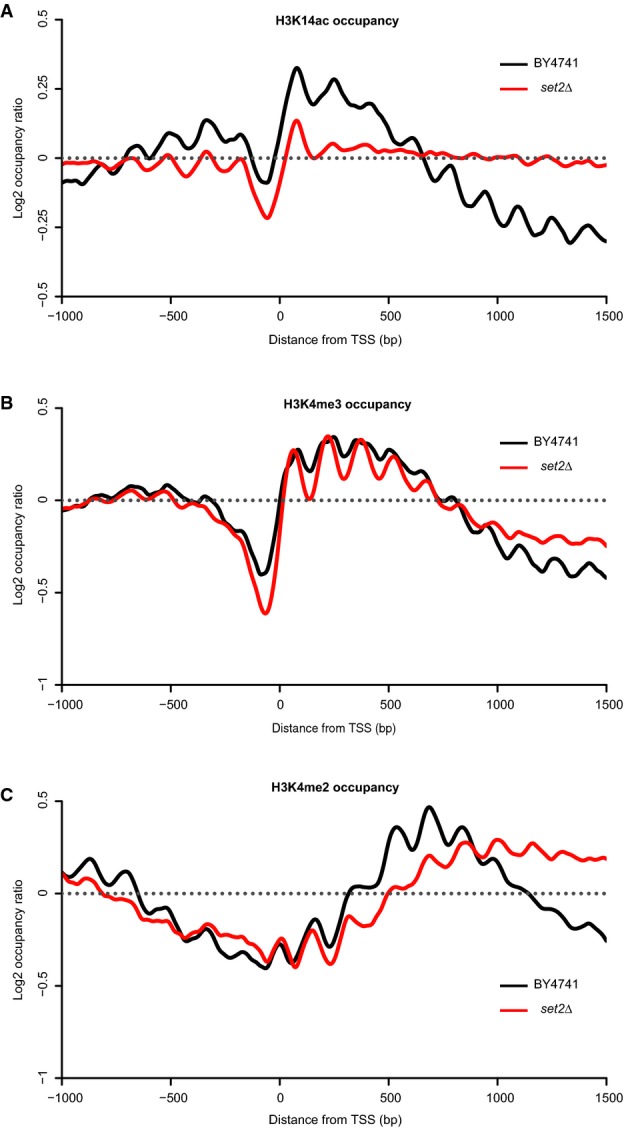
TSS plots confirming the patterns of histone marks previously observed in the *set2*Δ mutant, using a classical high-resolution ChIP-Seq protocol

Since signals for histone marks enrichment and consequently depletion tend to be exaggerated with Bar-ChIP as shown above in the experiment comparing our method to classical ChIP-Seq, distributions of H3K4me3, H3K4me2 and H3K4ac marks were independently corroborated for the wild-type and *set2*Δ strains with the latter approach. No difference in nucleosome occupancy was detected between the two strains (Supplementary Fig. S16). These data also confirmed the uniform distribution of H3K14 acetylation along gene bodies in *set2*Δ as opposed to the presence of this histone modification mostly in the 5′ region of genes in the wild-type strain (Fig5A; Supplementary Fig. S17A). Similar to what we observed with Bar-ChIP, both H3K4me3 and H3K4me2 marks were enriched near the 3′ end of annotated genes in the *set2*Δ mutant and these differences were statistically significant (Fig5B and C; Supplementary Fig. S17B and C).

H3K14ac (A), H3K4me3 (B) and H3K4me2 (C) occupancies in the wild-type strain and in the *set2*Δ mutant. Occupancies were normalized using counts from the chromatin inputs.

Previous work has shown that deletion of Set2p in yeast leads to an increase of spurious transcription within gene bodies, with the emergence of cryptic sites of transcription initiation (Carrozza *et al*, 2005; Li *et al*, 2007c). High-resolution tiling microarray studies have indicated that the phenotypic consequences of SET2 deletion vary across genes and that the appearance of new transcripts is restricted to a subset of genes (Li *et al*, 2007c). As H3K14ac and H3K4me3 marks are characteristic of highly active promoters, we assessed whether an increased occupancy for these marks is associated with the emergence of cryptic transcripts.

Analysis of a TSS dataset generated in our laboratory (Pelechano *et al*, manuscript in preparation), using a modified version of the Cap Analysis Gene Expression (CAGE) approach that permits unambiguous identification of TSS genome-wide (Pelechano *et al*, 2014), allowed us to identify genes harboring cryptic sites of transcription initiation in the *set2*Δ mutant. We detected more than 700 genes with increased internal transcription initiation (< 10% of examined genomic features, Supplementary Table S1), most of which were long genes (2,830 bp on average as compared to the genome-wide 1,500 bp average) as reported for a small set of genes in (Li *et al*, 2007c) and (Venkatesh *et al*, 2012) (Supplementary Figs S18 and S19).

**Figure 6:**
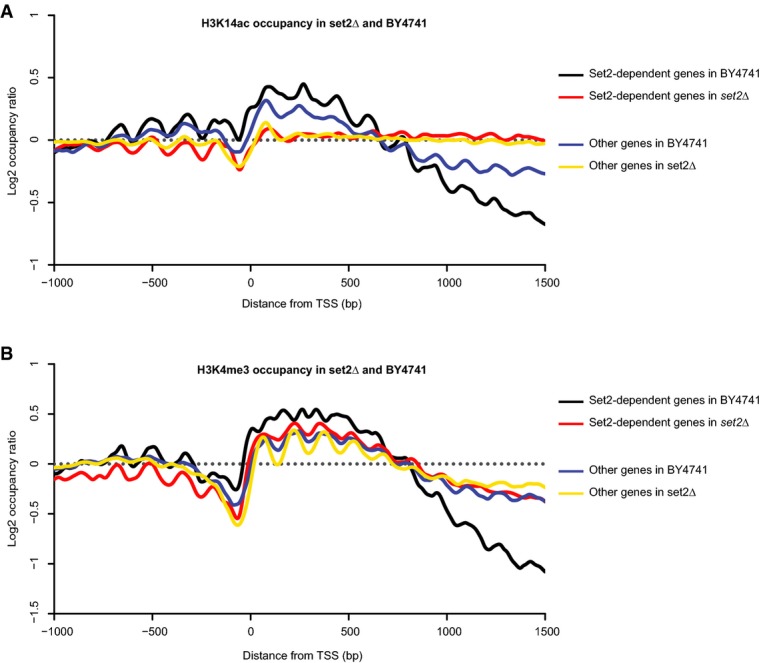
Distribution of H3K14ac and H3K4me3 for Set2-dependent and -independent genes in wild-type and *set2*Δ strains TSS plots representing the distribution of H3K14ac and H3K4me3 for genes grouped as either Set2-dependent or Set2-independent in the wild-type strain and *set2*Δ mutant.

Occupancy profiles of H3K14ac and H3K4me3 were then computed for this group of genes in both wild-type and mutant strains (Fig6; Supplementary Figs S20 and S21). As a control, we also analyzed patterns of these histone marks for the genes with no significant increase in internal transcription initiation. Surprisingly, H3K14ac levels in *set2Δ* remained elevated across the entire body of genes, regardless of the appearance of internal transcripts (Fig6A). In contrast, we observed a strong elevation of H3K4me3 occupancy toward the 3′ region of genes that showed emergence of cryptic transcripts (Set2-dependent genes) (Fig6B). Thus, increased H3K4me3 occupancy correlated with spurious transcription, while H3K14ac levels appeared to globally accumulate across the genome.

H3K14ac occupancies around the annotated TSS in two groups of genes in the BY4741 and *set2*Δ strains. Set2-dependent genes were identified using unambiguous genome-wide mapping of internal transcription start sites.

H3K4me3 occupancies around the annotated TSS in two groups of genes in the BY4741 and *set2*Δ strains. Data information: Set2-dependent genes are either in black (for BY4741) or red (for *set2*Δ), while Set2-independent genes are represented as a blue line (for BY4741) or yellow line (for *set2*Δ).

### Origin of internal H3K14 acetylation and H3K4 methylation in the *set2*Δ strain

Venkatesh *et al* (2012) previously suggested that enrichment of acetyl marks in the context of H3K9, H3K56 and H4 acetylation results from an increased histone turnover. We therefore tested whether the global accumulation in H3K14ac observed in *set2*Δ and the two Rpd3S-associated mutants (*rco1*Δ and *eaf3*Δ) may also originate from an increased incorporation of acetylated histones. To this end, we profiled the genome-wide distribution of these histone marks after disrupting histone exchange via the deletion of the histone chaperones, Asf1p and Rtt109p, in a *set2*Δ background. To only examine DNA replication-independent histone exchange, the mutant strains were profiled under arrested growth conditions with an efficiency ranging from 40 to 60%. No obvious difference in the H3K14ac profiles obtained for the double-mutants was detected in comparison to *set2*Δ, except for a modest effect observed in the *rtt109*Δ*set2*Δ*bar1*Δ mutant (Supplementary Fig. S22). In consequence, while H3K14ac appears to be maintained at low levels within gene bodies through the activation of Rpd3S, the mechanism of its incorporation in the *set2*Δ mutant does not seem to involve the classical histone exchange pathway.

Finally, to address a potential crosstalk between H3K14ac and H3K4me3/2 in the 3′ region of genes, we introduced mutations deleterious for histone H3 acetylation (Maltby *et al*, 2012) in a *set2*Δ background (*set2*Δ*ada2*Δ*sas3*Δ). The triple mutant did not display any striking difference in the genome-wide distribution of H3K4me3 in comparison to that obtained in *set2*Δ, while a substantial decrease in H3K4me2 was detected near the 3′ end of genes, about 1,000 bp downstream of the TSS (Supplementary Fig. S16). In contrast, abrogation of H3 acetylation resulted in an increased level of H3K4me2 in the intragenic region between the TSS and 500 bp downstream of the TSS, similar to that observed in *ada2*Δ*sas3*Δ (Supplementary Fig. S23). This enrichment in H3K4me2 in the double mutant has been reported elsewhere (Maltby *et al*, 2012) and shown to be linked to the histone demethylase, Jhd2p, whose activity on H3K4me3 is triggered by H3K14ac depletion (Liang *et al*, 2007; Maltby *et al*, 2012). Our results suggest that the interactions between H3K14ac and H3K4me3/2 toward the 3′ end of genes are of a distinct nature and may involve other molecular partners.

## Discussion

Chromatin immuno-precipitation followed by sequencing (ChIP-Seq) has provided major insights into the molecular mechanisms underlying chromatin dynamics and transcription regulation. In this study, we present Bar-ChIP, a method derived from ChIP-Seq that drastically accelerates its current experimental throughput without the need for robotics instrumentation. We compared the quality and precision of the data obtained using Bar-ChIP with those generated with traditional ChIP-Seq and assessed the multiplexing potential of the approach. Bar-ChIP led to several findings that shed light on the interplay between H3K14 acetylation and H3K4 methylation, which we further investigated. Results of these additional studies are also reported here.

Bar-ChIP relies on direct molecular barcoding of fragmented chromatin prior to immuno-precipitation, an approach, which, as we demonstrated, does not impact the nucleosomal distribution obtained after chromatin fragmentation. Furthermore, this new technology faithfully captures mono-nucleosome fractions that are enriched for a targeted histone mark. Indeed, genome-wide profiling of H3K4me3 in *S. cerevisiae* using Bar-ChIP recovered the distribution of nucleosomes marked with this histone modification, with a high inter-sample reproducibility, similar to that obtained with traditional ChIP. Absolute enrichments slightly differed between the two techniques. This may possibly stem from potential biases in both nucleosome barcoding and immuno-precipitation, given the somewhat longer nucleosome-protected fragments that are recovered with Bar-ChIP. Nevertheless, our observations indicate that the ligation of molecular adapters to fragmented chromatin does not interfere with the propensity to pull-down protein–DNA complexes using specific antibodies. By generating the equivalent of 90 distinct ChIP-Seq datasets within a few days, we demonstrate that the use of molecular barcodes permits the simultaneous immuno-precipitation of all biological samples as a pool of fragmented chromatin, hereby reducing the technical variability, which is inherent to experiments performed sequentially. The limited number of estimated unique molecules retrieved per IP reflects a moderate complexity of the sequencing libraries. This might originate from a limited efficiency of the nucleosome barcoding step, added to a possible preference of the ligase for certain populations of nucleosomes. Given the aforementioned potential biases and the modest resolution of the data, Bar-ChIP is an ideal approach to obtain a quick genome-wide overview of histone PTM enrichment patterns. Interesting trends may then be confirmed by complementary experiments.

During the preparation of this manuscript, Lara-Astiaso *et al* (2014) reported the indexing of sheared cellular chromatin isolated from dendritic cells before immuno-precipitation. This independent work also highlights the advantage of such an approach to assay multiple samples simultaneously and insure inter-sample reproducibility. Our study demonstrates that the ligation of DNA adapters may be equally performed on nucleosomal fractions isolated by enzymatic treatment without the need for chromatin immobilization prior to the indexing. This confers single-nucleosome resolution and further increases the speed of the assay.

Bar-ChIP applied to genome-wide profiling of several histone modification mutants revealed striking differences in nucleosome positioning around the transcription start sites (TSS). *eaf3*Δ and *set1*Δ mutants displayed a nucleosome-depleted region 50 bp wider than that of the wild-type strain, as well as shorter +1 nucleosomes, indicative of a greater sensitivity to MNase. This suggests that the two mutants possibly display a more open chromatin structure or are associated with the presence of partially unwrapped nucleosomes or subnucleosomal particles (Henikoff *et al*, 2011). Additionally, while the multiplexing experiment confirmed the complete abrogation of H3K4 methylation in *set1*Δ, it also showed that this impacted the genome-wide pattern of H3K14ac and H3K36me3. In fact, both histone marks appeared to be enriched at the −1 nucleosome in comparison to the wild-type strain, which could represent disequilibrium in bidirectional promoter activity as previously observed in the context of histone hyperacetylation (Schuettengruber, 2002). Further investigation will be needed to assess whether such a similar mechanism plays a role in the localized PTM enrichment we observed in *set1*Δ.

Set2p, Rco1p and Eaf3p are all necessary for the activity of the Rpd3S complex which maintains low levels of acetylation within coding regions and prevents cryptic transcript initiation (Carrozza *et al*, 2005; Keogh *et al*, 2005; Drouin *et al*, 2010). Consistent with this model, analysis of *set2*Δ, *rco1*Δ and *eaf3*Δ profiles using Bar-ChIP revealed H3K14ac enrichment in the 3′ region of genes, with an accumulation of the mark throughout the entire body of genes. Genome-wide increased acetylation of histone H4, H3K9 and H3K56 was previously reported in yeast strains deleted for these proteins (Drouin *et al*, 2010; Venkatesh *et al*, 2012). This is the first time, however, that such a trend is specifically observed at the genome-wide level for H3K14. As the Rpd3S complex acts downstream of Set2p in the transcriptional elongation process through its recruitment and activation by H3K36me3 (Govind *et al*, 2010), our results imply that H3K14ac is also generally maintained at a low level within gene bodies via activity of the Rpd3S histone deacetylase complex.

Additionally, the *set2*Δ mutant displayed a significant enrichment for H3K4me3 and H3K4me2 in regions near the 3′ end of genes, which contrasted with the usual presence of these marks around promoters and just downstream of TSS, respectively (Ng *et al*, 2003; Pokholok *et al*, 2005; Li *et al*, 2007a). We showed that *set2*Δ atypical H3K4me3 pattern is mostly driven by genes with cryptic sites of transcription initiation, which display dramatic elevation in H3K4me3 occupancy at their 3′ end. This indicates a direct association between the appearance of the H3K4me3 mark in the body of genes and the emergence of spurious transcripts in the same genomic regions. Furthermore, our data imply that accumulation of H3K14ac in *set2*Δ derives from a process that is distinct from increased histone turnover, in contrast to acetylation of histone H4, H3K9 and H3K56 (Venkatesh *et al*, 2012). In comparison, when analyzing *set2*Δ in combination with ada2Δ*sas3Δ*, H3K4me3 was not affected while increased levels of H3K4me2 were observed between the TSS and 500 bp downstream of the TSS, with a substantial depletion of the mark toward the 3′ region of genes. This suggests that, while H3K14 acetylation may negatively regulate H3K4me3 demethylation by the histone demethylase Jhd2p in gene bodies (Maltby *et al*, 2012), the possible interplay between H3K14 acetylation and H3K4 methylation toward the 3′ region of genes relies on a different molecular mechanism.

In conclusion, we demonstrate that Bar-ChIP provides fast and accurate overview of the genome-wide chromatin status of multiple samples at once. Bar-ChIP faithfully captures patterns of histone marks and the sensitivity of the technique enables the detection of fine differences in histone modifications harbored by yeast chromatin-associated mutants.

## Materials and Methods

### Yeast strains, culture and crosslinking conditions

All *S. cerevisiae* strains used in this study are derived from BY4741 (Winzeler, 1999) and listed in Supplementary Table S2. The deletion strains were generated using standard yeast chemical transformation procedures (Gietz & Schiestl, 2007). The *ada2*Δs*et2*Δ strain was obtained by deletion of *SET2* and its replacement by *HIS3* in the BY4741*ada2*Δ background, while the *set2*Δ*ada2*Δ*sas3*Δ strain was constructed by replacing, in the BY4741*sas3*Δ background, *ADA2* and *SET2* with *URA3* and *HIS3*, respectively.

Cells were grown in 50 ml YPD (1% yeast extract, 2% peptone, 2% glucose and 40 mg/l adenine) and harvested at an OD_600_ of ∽1. Crosslinking was performed with 1% formaldehyde (final concentration) for 15 min at room temperature on a shaker plate. Fixation was quenched with the addition of glycine to a final concentration of 0.135 M. Crosslinked cells were washed three times in ice-cold TBS buffer (20 mM Tris pH 7.5, 0.146 M NaCl). Pellets were flash-frozen in liquid nitrogen and stored at −80°C for later use.

### Nuclear extraction and nucleosomes barcoding

Yeast pellets were resuspended in NP-S buffer (0.5 mM spermidine, 0.075% NP-40, 10 mM Tris-HCl pH 7.4, 50 mM NaCl, 5 mM MgCl_2_, 1 mM CaCl_2_ and 1 mM β-mercaptoethanol) with Protease Inhibitor Cocktail (Roche) and lysed at 4°C with 2 volumes of glass beads in a FastPrep instrument (MP Biomedicals) using the following settings: 4 × 20 s at 6.5 m/s with 1 min pause between each pulse. Nucleosome fractions were isolated by incubating lysates with 80 U of micrococcal nuclease (Worthington Biochemicals) for 45 min at 37°C. EGTA was added to samples at a final concentration of 0.01 M for enzyme inactivation. Note that given the stronger affinity of EGTA for Ca^2+^ than Mg^2+^ ions, this concentration is sufficient to inactivate the MNase without inhibiting any of the downstream enzymatic reactions. MNase digestion generally produced about 80% of mono-nucleosomes as confirmed on a bioanalyzer (Agilent).

Barcoding of nucleosome fractions was performed using 50 μg of starting material as measured by absorbance at 260 nm with Nanodrop. Chromatin fragments were end-repaired with 2 μl of NEBNext End repair Enzyme Mix (New England BioLabs), dA-tailed with 2 mM dATP and 20 U Klenow Fragment (Thermo Scientific) and ligated for 4 h at 16°C to paired-end adapters containing a 6-mer multiplex barcode with 1,600 U T4-DNA ligase (New England BioLabs). Enzymes were heat-inactivated at 65–70°C between each step. Inactivation of the DNA ligase by incubating samples at 65°C for 15 min after the adapter ligation step was particularly important to prevent any further barcoding during the sample pooling and thus any cross-contamination between samples and adapters (Supplementary Fig. S24). Note that the length of incubation was not sufficient to promote reverse crosslinking of the samples, usually obtained after 12–14 h. Barcoded chromatin samples corresponding to the various wild-type and mutant yeast strains and replicates were pooled together in estimated equal amount before aliquoting the resulting pool for the diverse immuno-precipitation experiments.

### Chromatin immuno-precipitation

All antibodies used in the study are listed in Supplementary Table S3.

Three microliters of anti-H3K4me3, 10 μl of anti-H3K4me2, 10 μl of anti-H3K4me1, 5 μl of anti-H3K14ac and 4.5 μl of anti-H3K36me3 were used for each immuno-precipitation.

Antibodies were diluted in PBS containing 5 mg/ml BSA (Sigma) and 10–30 μg of yeast tRNA (Ambion) and conjugated to 1.5 and 4.5 mg of protein A Dynabeads (Invitrogen) for classical ChIP and Bar-ChIP, respectively, for 4 h at 4°C on a wheel. A no antibody control (i.e. protein A Dynabeads only) was included in all IP experiments.

One hundred and fifty micrograms of barcoded (Bar-ChIP) or 50 μg of non-barcoded nucleosomes (classical ChIP) were diluted in lysis buffer (50 mM Hepes, 140 mM NaCl, 1 mM EDTA, 1% Triton, 0.1% sodium deoxycholate, 1 mM benzamidine and Protease Inhibitor Cocktail) and incubated with antibody-coated magnetic beads overnight at 4°C on a wheel. Chromatin–antibody complexes were washed eight times in RIPA buffer (0.05 M Hepes, 0.5 M LiCl, 1 mM EDTA, 1% NP-40 and 0.7% sodium deoxycholate), and chromatin was eluted from the antibody-coated beads by two 10-min incubations in ChIP elution buffer (50 mM Tris pH 8.0, 10 mM EDTA and 1% SDS) at 68°C with mixing.

Crosslinking was reversed by incubating chromatin with 1 μl RNase cocktail (Ambion) for 1–2 h at 37°C, and degrading proteins with 150 μg (3 U) proteinase K (Invitrogen) for 14 h at 65°C.

IP DNA and input DNA were purified with Ampure XP magnetic beads (Agencourt) according to the manufacturer's instructions and diluted in 10 mM Tris–HCl pH 8.5.

IP enrichments were also confirmed by quantitative PCRs (see below).

### Sequencing library preparation

For classical ChIP experiments, 20–100 ng of IP DNA and input DNA were end-repaired, dA-tailed and ligated to paired-end adapters as described above. Purification of the newly barcoded DNA samples was performed using Ampure XP beads.

One nanogram of barcoded IP DNA and input DNA from Bar-ChIP and classical ChIP experiments was amplified by PCR using 0.4 μl of each Illumina paired-end primers (at 10 μM) and 20 μl of 2× Phusion HF Master Mix (New England Biolabs) with the following program: 30 s at 98°C for initial denaturation, 30 s at 98°C, 30 s at 65°C, 30 s at 72°C for 18 cycles, followed by 5 min at 72°C for final extension. PCR products were purified using Ampure XP beads, and selection of 300 bp fragments was carried out with SPRI select beads (Beckman Coulter Life Sciences) following the manufacturer's instructions.

Libraries were sequenced on the Illumina HiSeq 2000 system using 105 bp paired-end reads.

### Quantitative PCR assays

For quantitative PCR assays, IP DNA and input DNA were used at 1/10 and 1/50 dilutions, respectively, while DNA from control IP (no antibody) was used undiluted. Amplifications were performed with primers spanning the ARO1 locus using Sybr Green PCR Master Mix (Applied Biosystems). Primers were designed based on the study of Pokholok *et al* (2005) for identification of regions positive and negative for the probed histone marks. Primers for the ARO1 region positive for H3K4me3, H3K4me2, H3K4me1 and H3K14ac were as follows: forward, 5′-ACGCCGACTCGTAACCATTA-3′; reverse, 5′-TGGCTAACTGCACCATCGTA-3′, while primers for the ARO1 region positive for H3K36me3 were as follows: forward, 5′-ACGTCATTGAAAGTGATGCCT-3′; reverse, 5′-GCATCACCTTGTAACGACTCA-3′. Primers for the ARO1 region negative for all these marks were as follows: forward, 5′-AACTTCGAAATTGAAGCCATT-3′; reverse, 5′-ACGCCAACGTGTTCCTTAAT-3′. The following qPCR conditions were used: 2 min at 50°C, 10 min at 95°C, followed by 40 cycles of 15 s at 95°C and 1 min at 60°C. All primers were used at a final concentration of 300–500 nM.

### Sequence alignment and read filtering

Illumina HiSeq 94 bp sequencing tags were aligned to the S288c reference genome (*S. cerevisiae* version R64-1-1) using Bowtie2 software (version 2.1.0) with no mismatch allowed within the seed alignment (seed of length 22, default setting). Each aligned dataset was then filtered for read pairs with a unique, unambiguous alignment and in convergent orientation (inward-facing read pairs). Pairs of reads defining DNA fragments with a size greater than 220 bp and therefore assumed to not correspond to a mono-nucleosomal fraction were filtered out. Selection of this size threshold was based on the profiles obtained for the chromatin samples run on a bioanalyzer, suggesting that mono-nucleosome sizes ranged from 148 to 152 bp. Additionally, for the aligned datasets derived from the multiplexing experiment, paired-end reads defining DNA fragments with a size smaller than 130 bp were also removed. Finally, estimated PCR duplicates (read pairs defining DNA fragments with more than two identical copies with the same first and last genomic positions) were also filtered out.

### Track visualization and TSS plots

Nucleosome prediction was based on the assumption that each read pair spanned the length of a single-nucleosome molecule and could be used to define its position. The midpoint of the nucleosome was then approximated as being the center of the genomic locus intercepted by the read pair. To visualize local nucleosome occupancy, we determined the number of nucleosome molecules located at each genomic position and used the IGB browser to view the associated coverage tracks.

To determine the global number of nucleosomes at each position around the annotated transcription start sites, we used an updated transcript annotation from Xu *et al* (2009) and the HTSeq package (Anders *et al*, 2014). These counts were then summed across all genes and divided by the total number of observed nucleosomes, thereby providing the genome-wide distribution of nucleosomes at each position around transcription start sites (TSS). This distribution was used to assign an occupancy level to each position around the TSS. Occupancy levels were plotted as a function of the distance to the TSS. Such plots were designated as TSS plots.

The occupancy ratio for a given histone mark was computed as the ratio of the occupancies derived from both input nucleosomal DNA and IP DNA. This ratio was plotted as a function of the distance to the TSS. To take into consideration the variations in MNase sensitivity between the profiled yeast strains and therefore the difference in the size of the chromatin fragments, notably between *set1Δ* and *eaf3Δ* mutants and all other strains (Supplementary Fig. S11), an extended window size for mono-nucleosome fragments was used to generate nucleosome counts for the inputs (to include fragments as small as 100 bp). Use of the same window size for the IP fractions did not, however, modify the computation of histone occupancies (Supplementary Fig. S25).

### Statistical analysis of the histone mark distribution

To compare histone mark distributions between two strains or conditions, we used the DESeq2 package (Love *et al*, 2014), which allows for both a normalization of the nucleosome counts using the chromatin input datasets and an estimation of the biological noise thanks to the biological replicate information. For each replicate and each gene, we computed the number of nucleosomes at every position around the TSS, both in the dataset generated from the IP and in the chromatin input. After removal of null counts, we estimated the average number of nucleosomes per gene at each position around the TSS—this number was computed for both the input and IP datasets. These counts were then used to call for significant differences using the DESeq2 package. Nucleosome counts from the chromatin inputs served as normalization factors, after being pondered by the coverage ratio between input and the datasets. These factors are used to correct for potential technical biases such as chromatin solubility, enzyme accessibility or preferential PCR amplifications. Local FDR values were computed from the *P*-values returned by DESeq2 using the fdrtool package (local FDR threshold was set to 0.2) (Bernd Klaus and Korbinian Strimmer, 2014). Finally, only the positions with a log2 fold change greater than 0.05 and a local FDR value smaller than 0.2 were considered significant. To choose such a threshold for fold change, we compared two sets of biological triplicates of H3K4me3 profiles generated at an interval of several months. We assumed that the threshold should be such that we do not observe any differences between those datasets and therefore selected the 0.05 value (Supplementary Fig. S5). This threshold was then applied to all further comparisons. Results were plotted using the ggplot2 package. Mean normalized counts per replicates were computed using the smhuber function from the smoothmest package (Christian Hennig, 2014).

### Call for Set2-dependent genes

Sequencing reads were aligned to the S288c reference genome (*S. cerevisiae* version R64-1-1) using Novoalign software with default settings. Reads with multiple alignments and/or carrying soft-clipped bases on the 5′ end or on more than 10% of the total read length were excluded from downstream analysis. We used the HTSeq package to evaluate the number of reads whose 5′ end fell within an annotated gene feature, after exclusion of the first 200 bp, hereby narrowing down the effect of Set2 on the 3′ portion of annotated genes. The resulting count tables were then used to call for differentially expressed genes using the DESeq2 package. The standard analysis workflow was used as described in the vignettes. Genes with a log2 fold change greater than 0 and an adjusted *P*-value smaller than 0.1 were considered to be Set2 dependent as they gave rise to an increased number of internal transcription initiation sites in the *set2*Δ mutant.

### Data availability

All ChIP-Seq and Bar-ChIP data can be accessed through the ENA accession number ERP007035.

Transcriptomic data corresponding to the profile for BY4741 and *set2Δ* are accessible through the GEO accession number GSE62735.

## Supporting Information

Supplementary Table S3

Review Process File
