# Supplementary figures and images for: A high-throughput ChIP-Seq for large-scale chromatin studies
Source: Mol Syst Biol. 2015 Jan 12;11(1):777. doi: 10.15252/msb.20145776 (PMC4332152; doi:10.15252/msb.20145776)

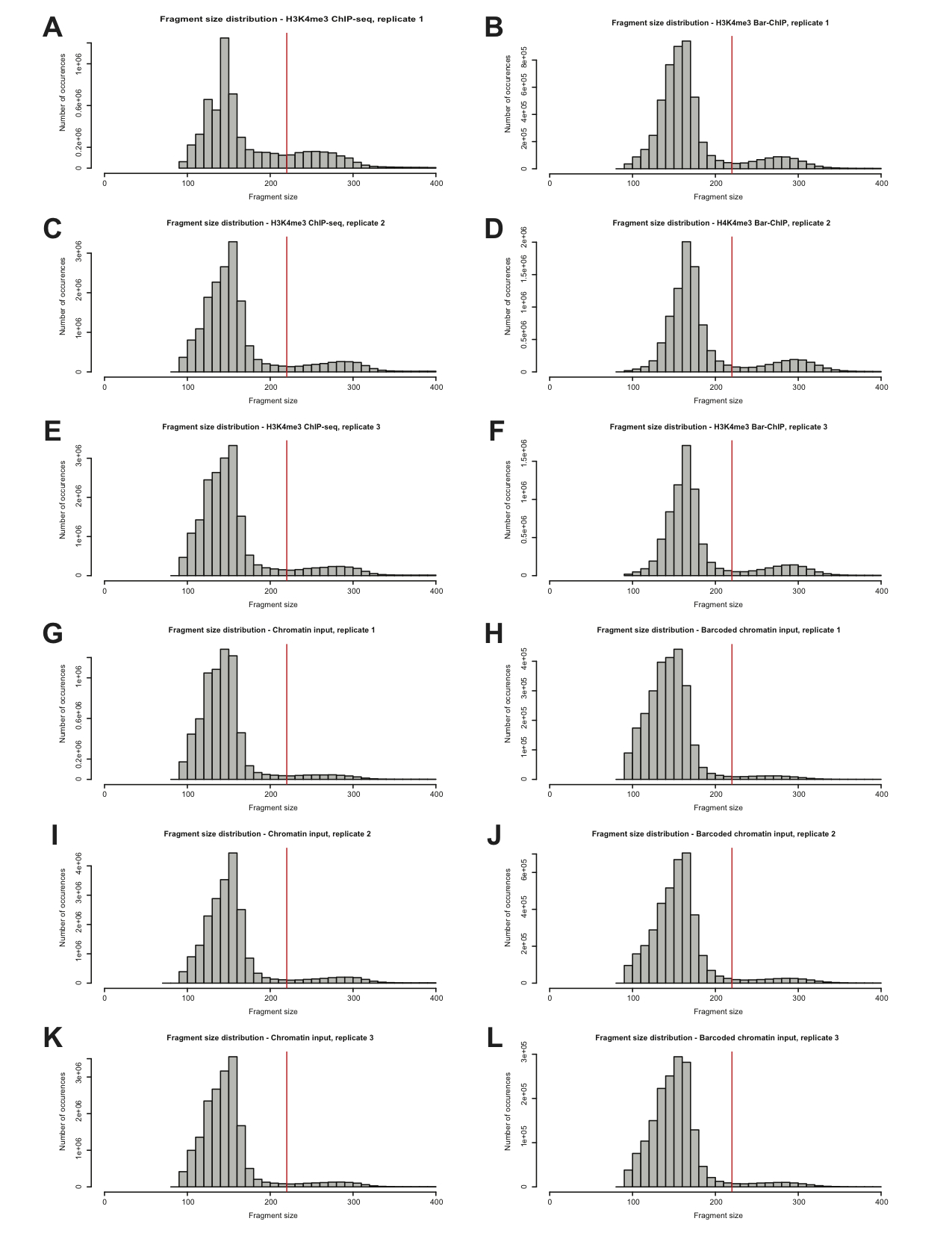

Supplement: Supplementary file 1 [file msb0011-0777-sd1.jpg]

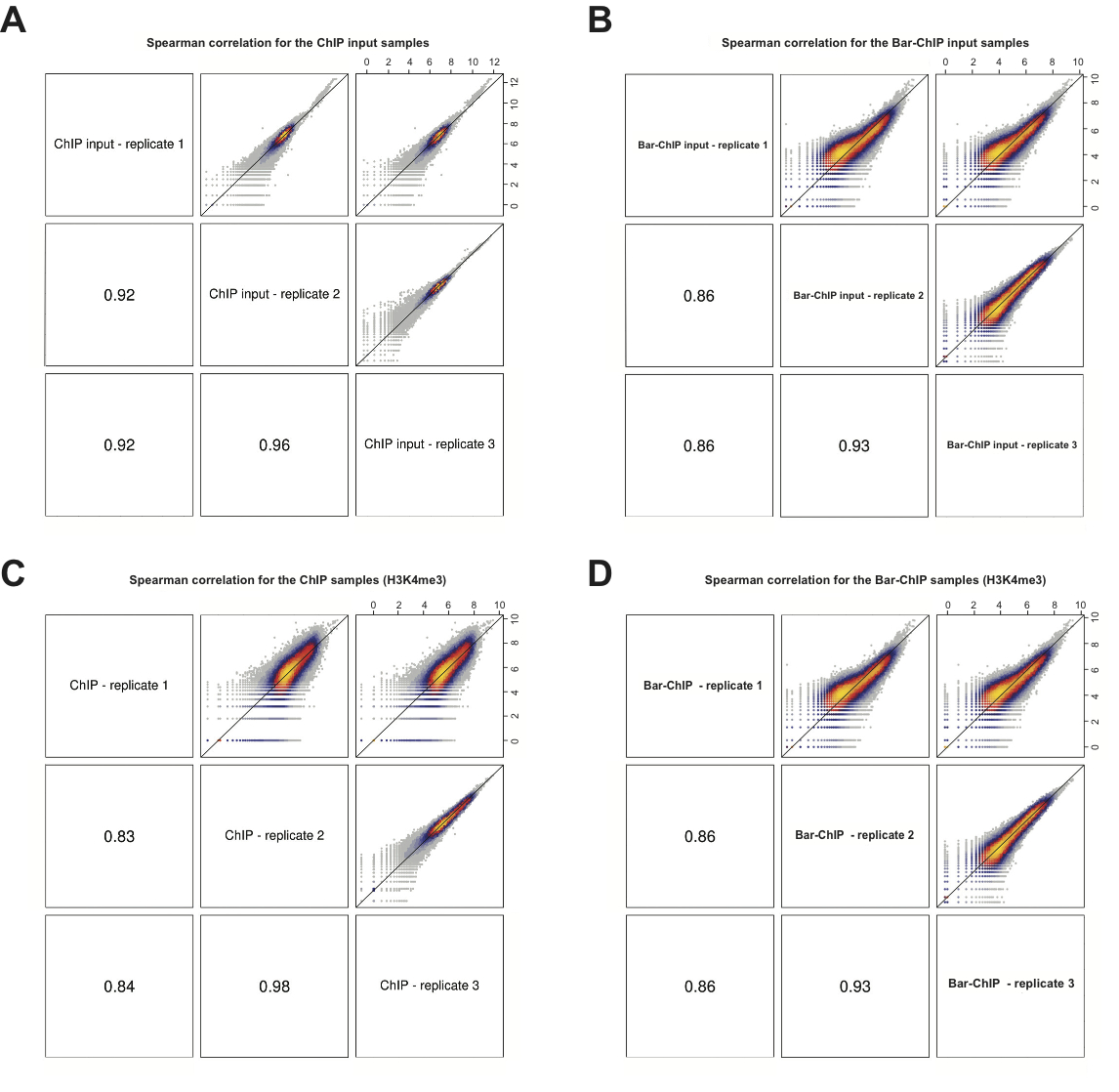

Supplement: Supplementary file 2 [file msb0011-0777-sd2.jpg]

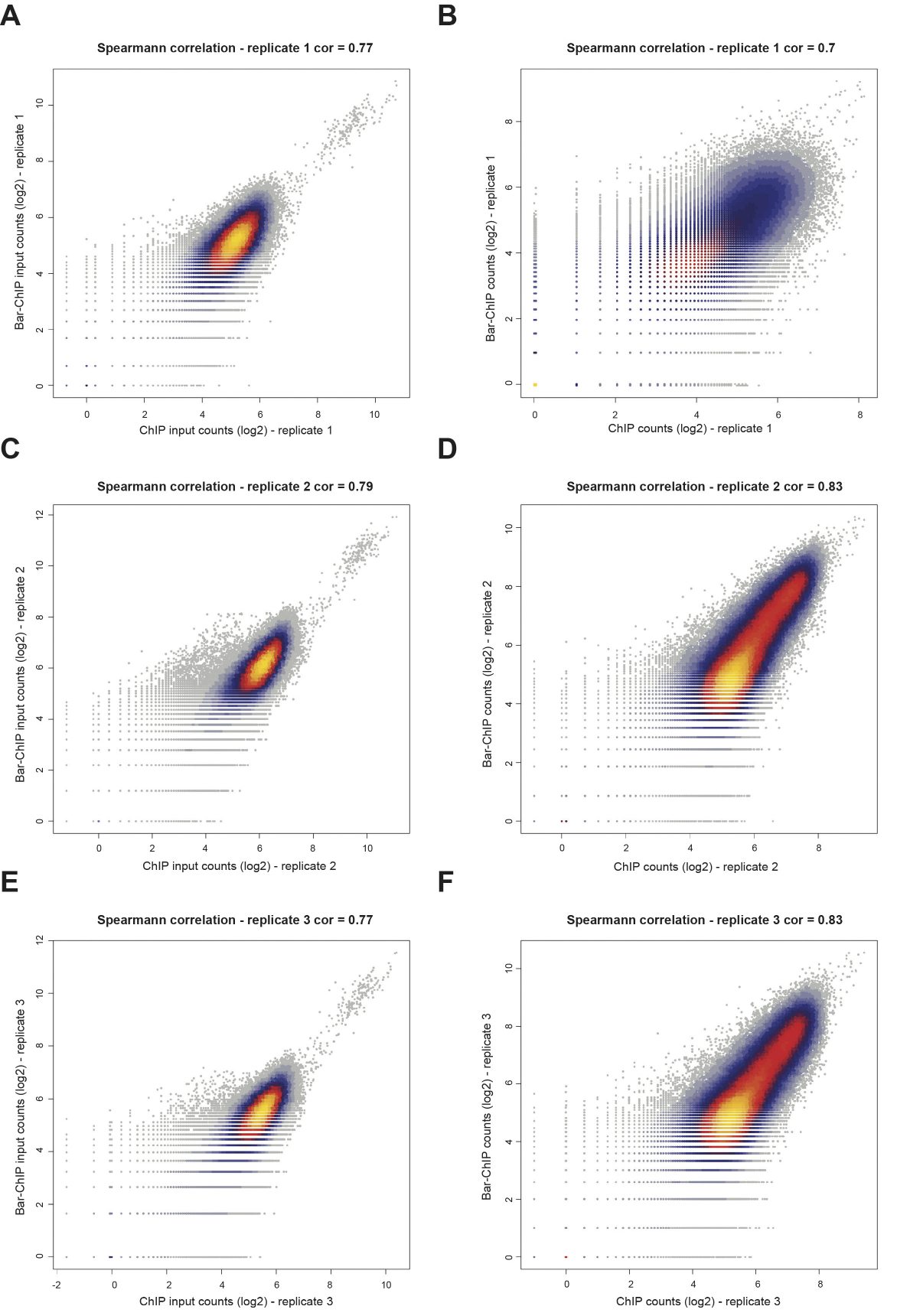

Supplement: Supplementary file 3 [file msb0011-0777-sd3.jpg]

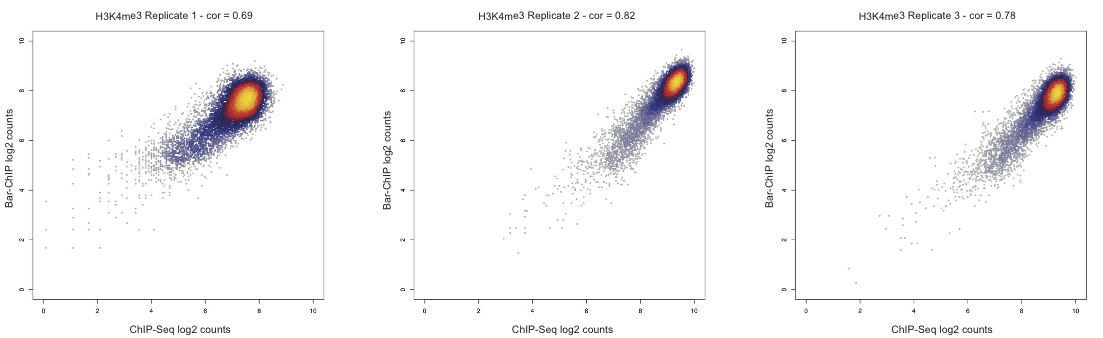

Supplement: Supplementary file 4 [file msb0011-0777-sd4.jpg]

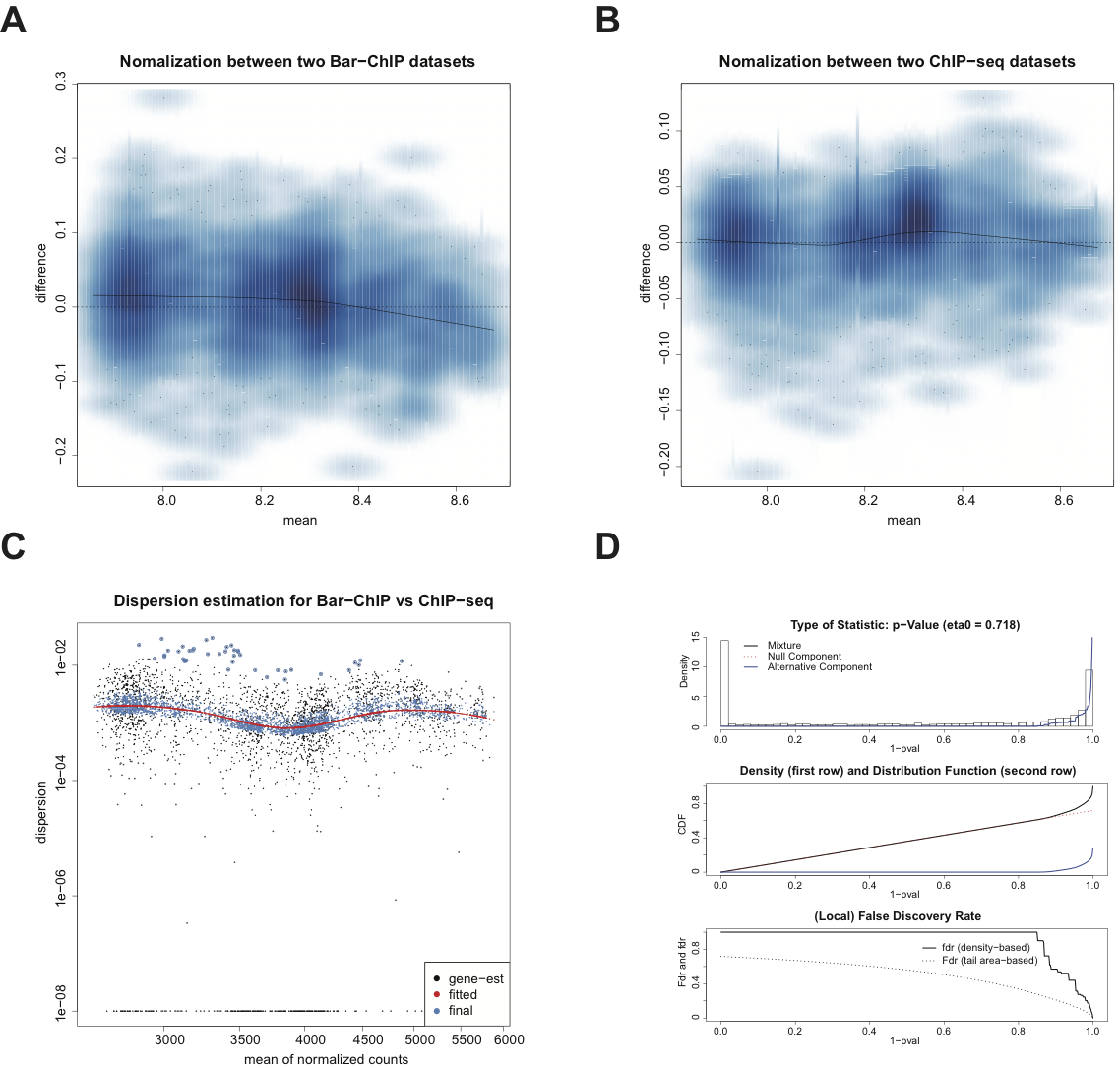

Supplement: Supplementary file 5 [file msb0011-0777-sd5.jpg]

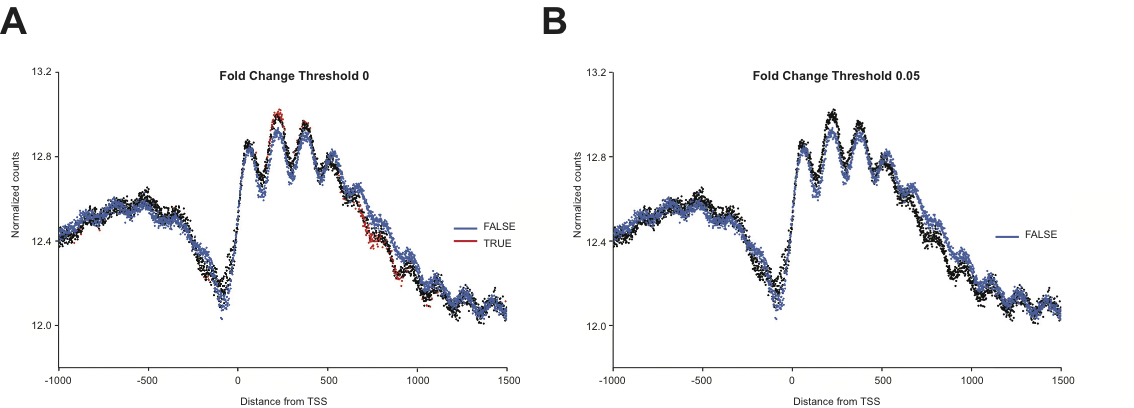

Supplement: Supplementary file 6 [file msb0011-0777-sd6.jpg]

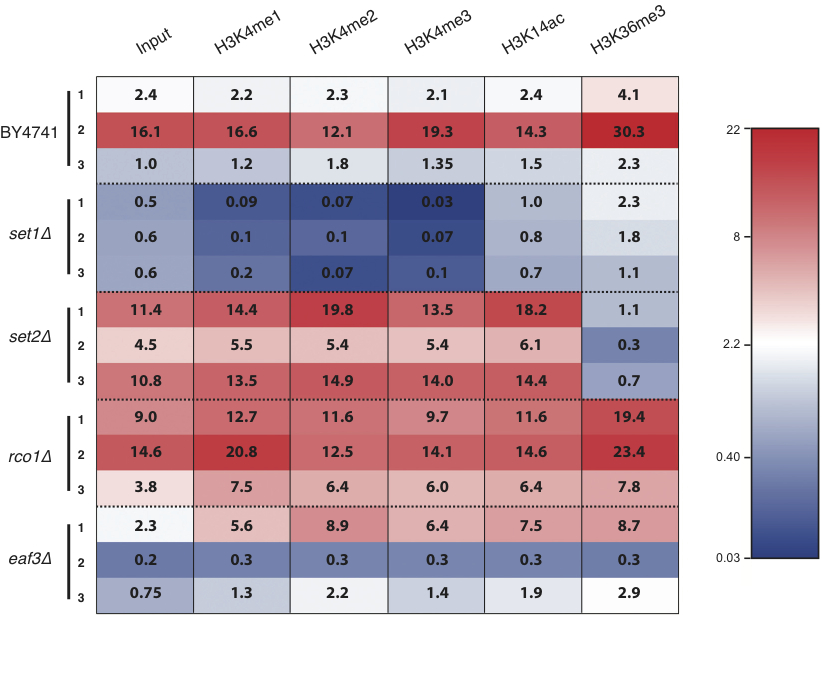

Supplement: Supplementary file 7 [file msb0011-0777-sd7.jpg]

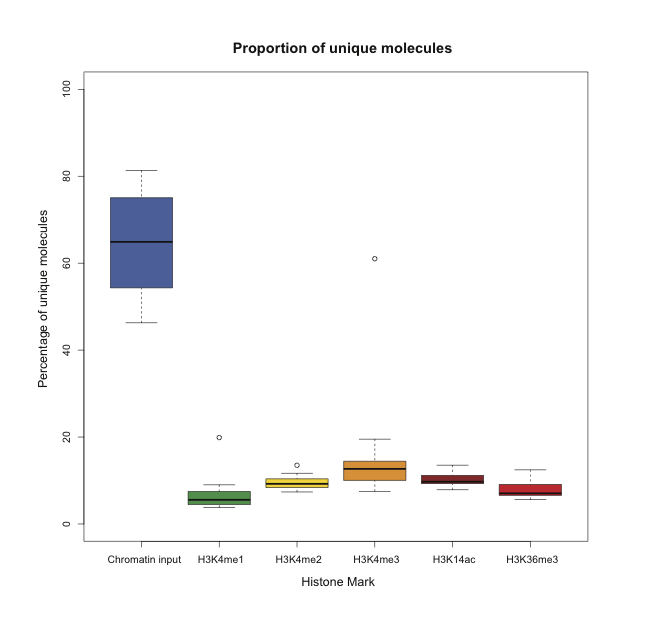

Supplement: Supplementary file 8 [file msb0011-0777-sd8.jpg]

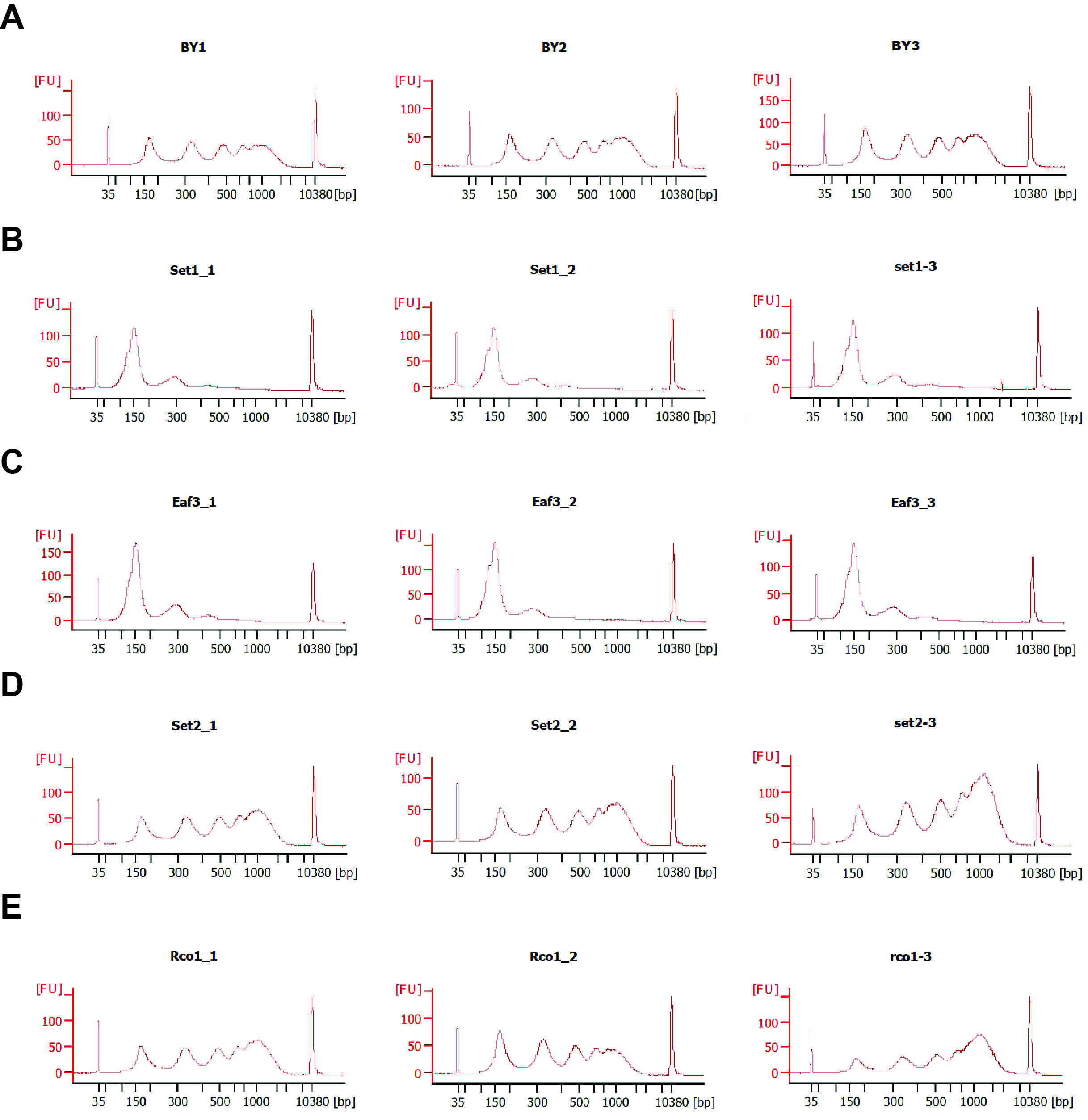

Supplement: Supplementary file 9 [file msb0011-0777-sd9.jpg]

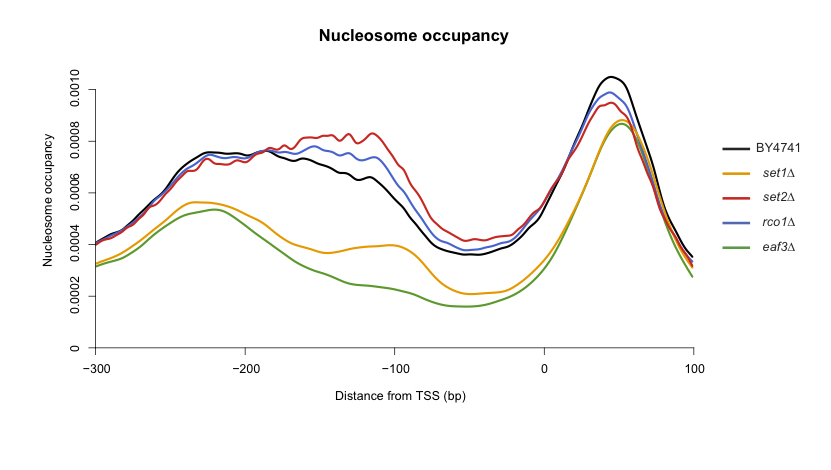

Supplement: Supplementary file 10 [file msb0011-0777-sd10.jpg]

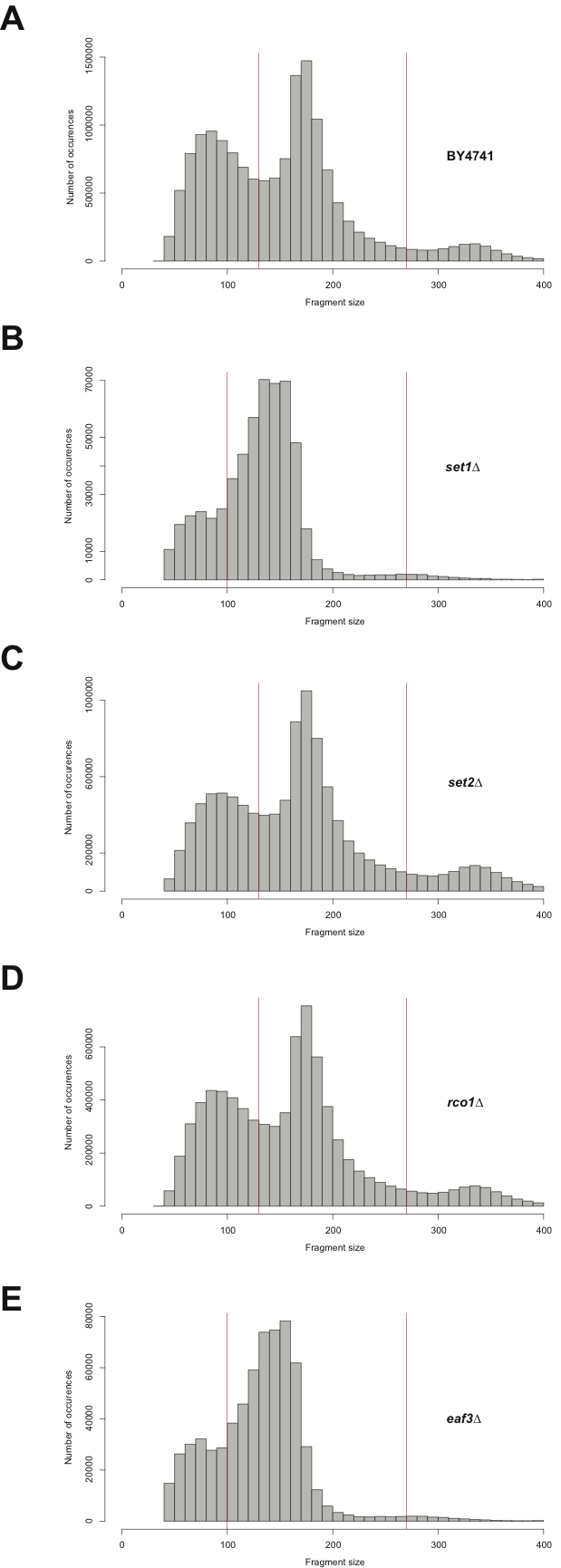

Supplement: Supplementary file 11 [file msb0011-0777-sd11.jpg]

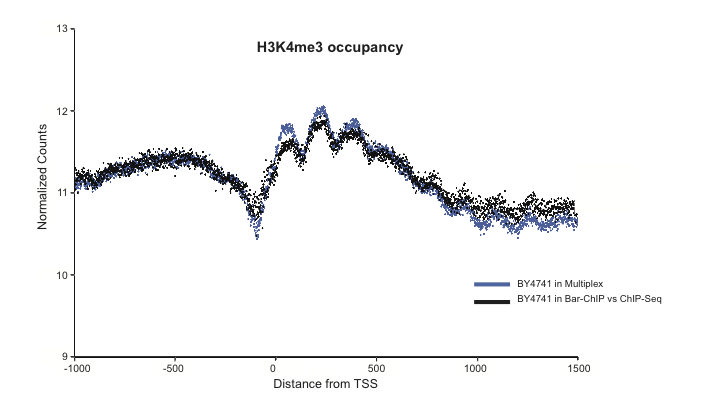

Supplement: Supplementary file 12 [file msb0011-0777-sd12.jpg]

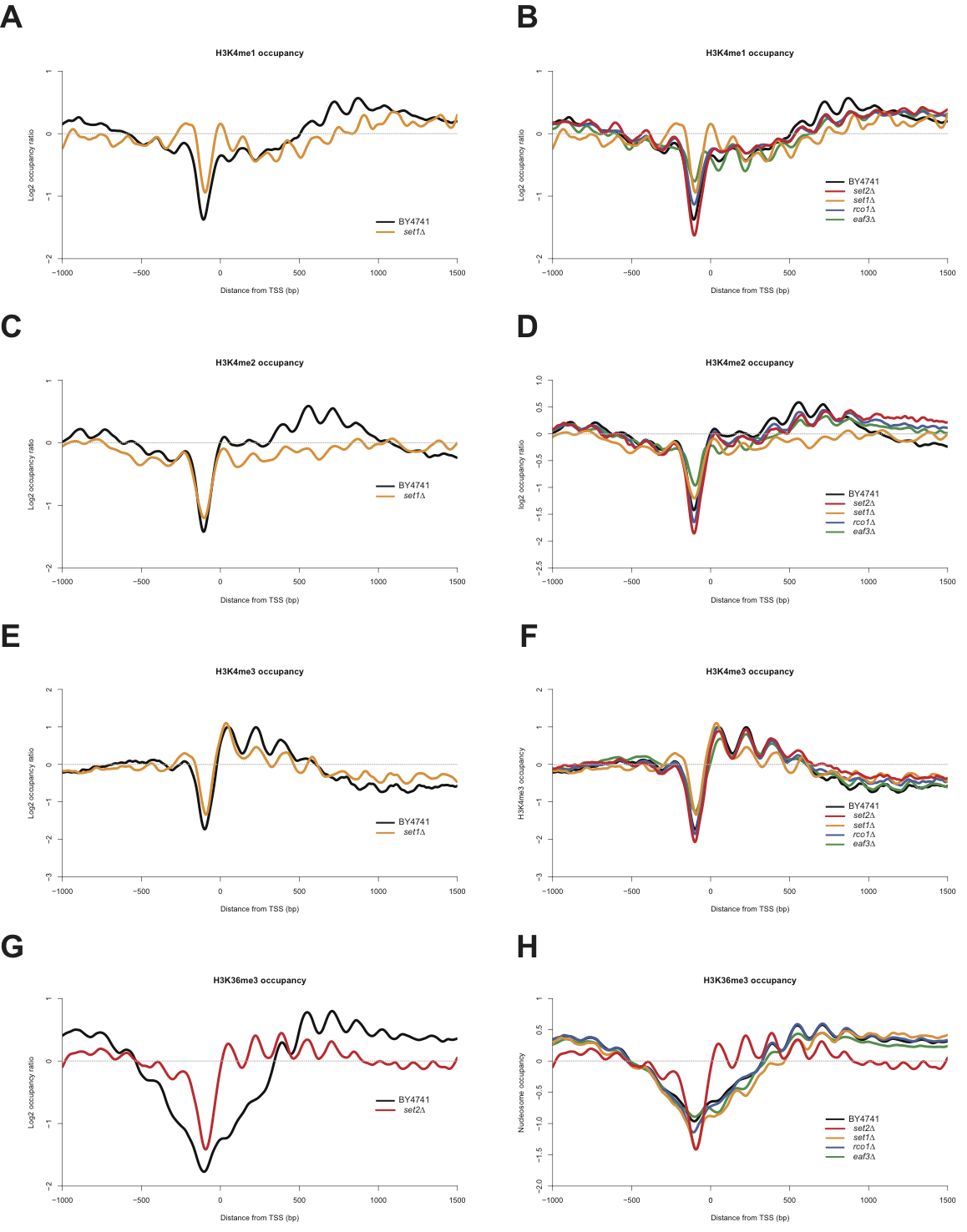

Supplement: Supplementary file 13 [file msb0011-0777-sd13.jpg]

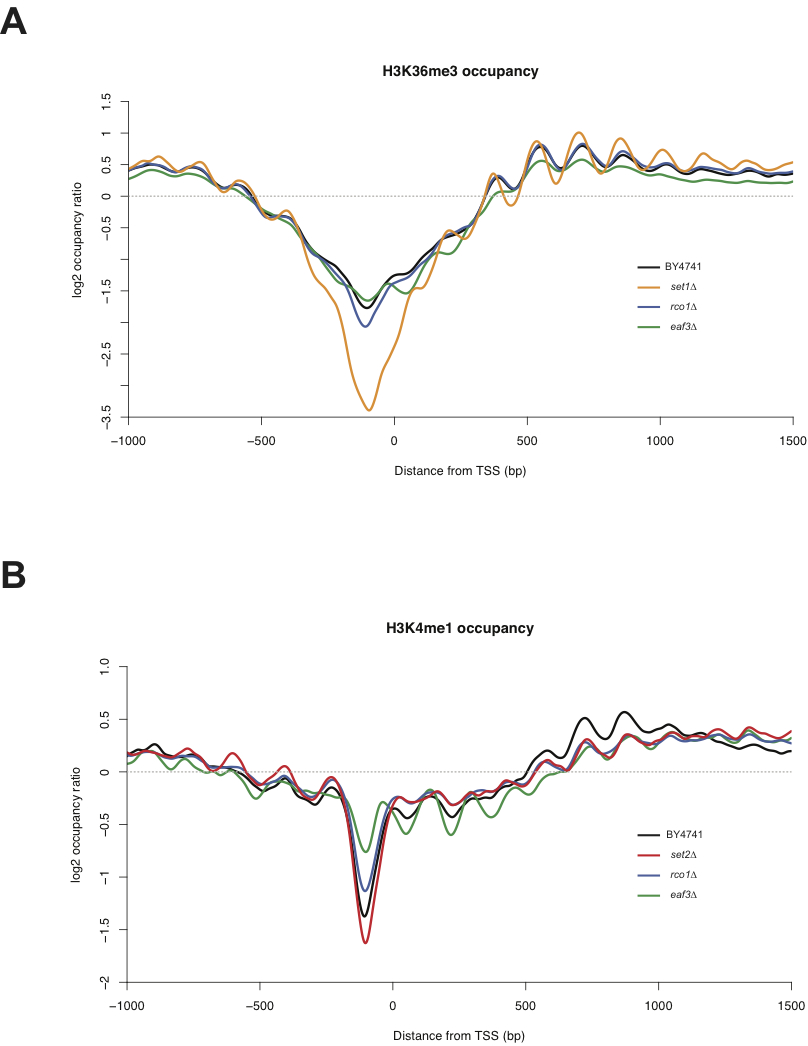

Supplement: Supplementary file 14 [file msb0011-0777-sd14.jpg]

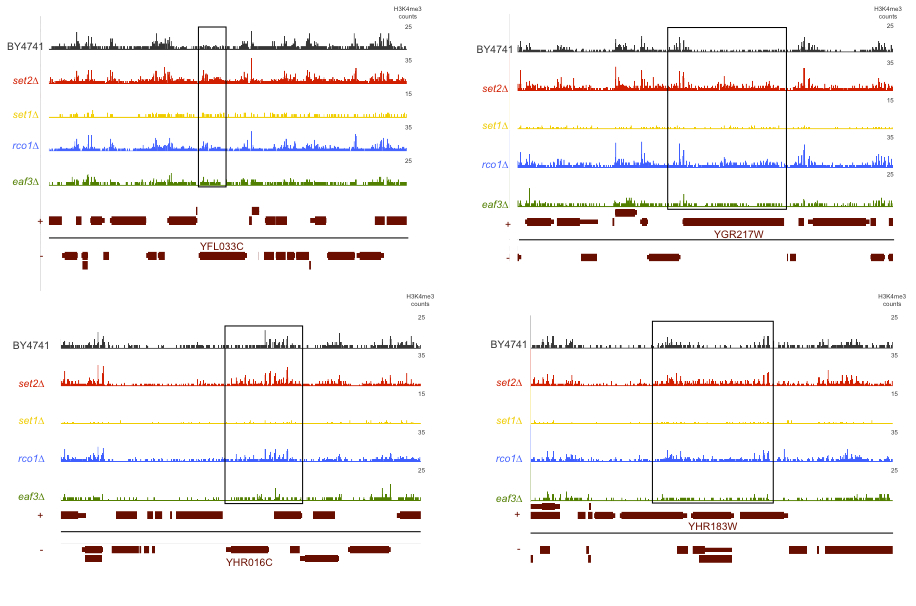

Supplement: Supplementary file 15 [file msb0011-0777-sd15.jpg]

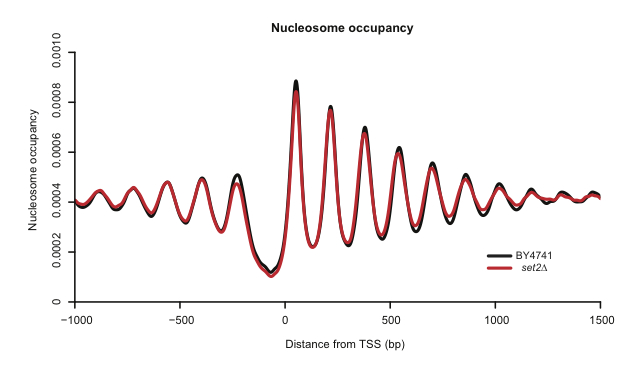

Supplement: Supplementary file 16 [file msb0011-0777-sd16.jpg]

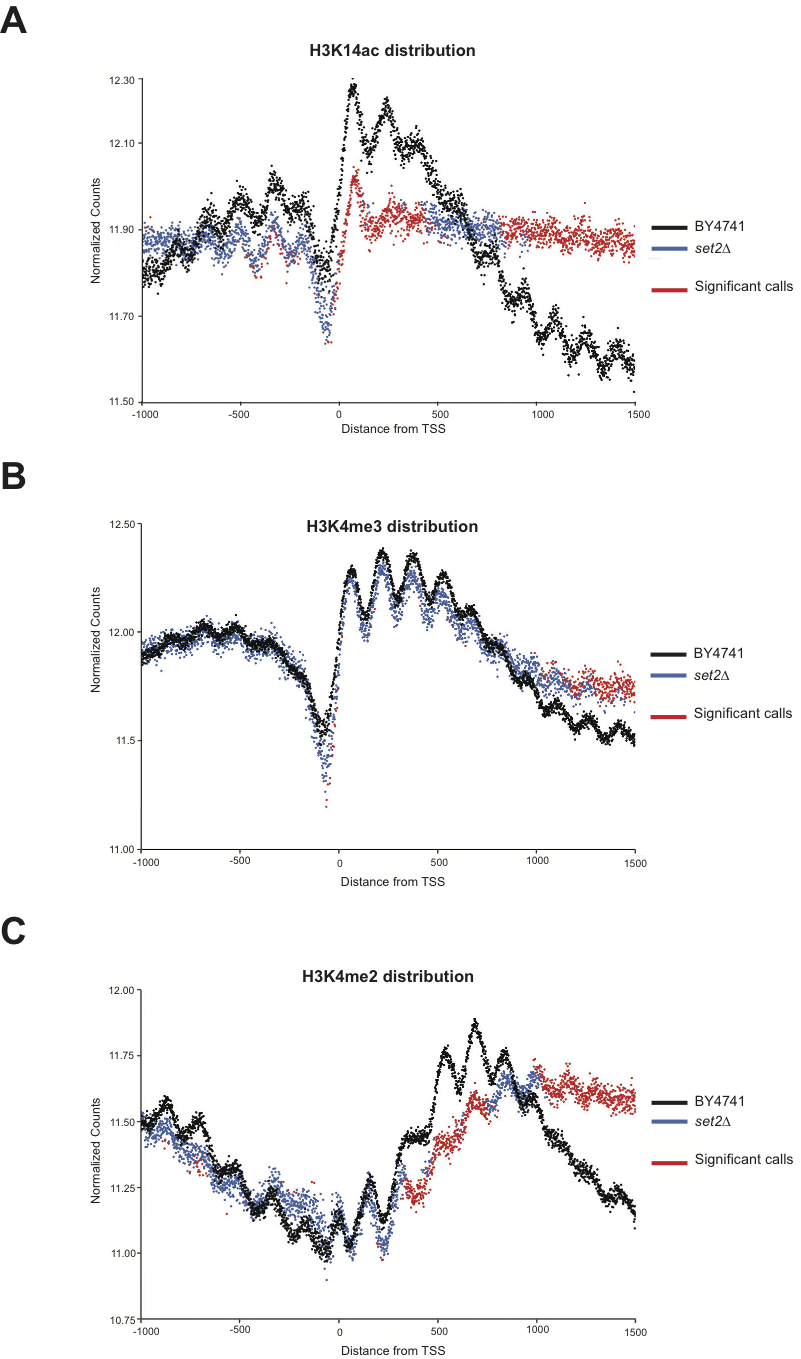

Supplement: Supplementary file 17 [file msb0011-0777-sd17.jpg]

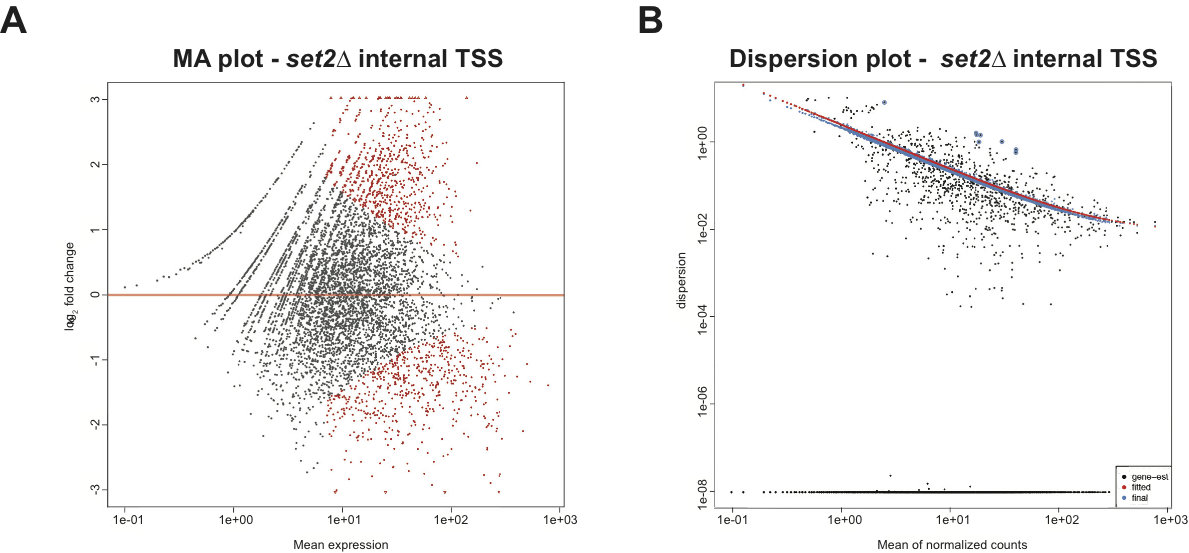

Supplement: Supplementary file 18 [file msb0011-0777-sd18.jpg]

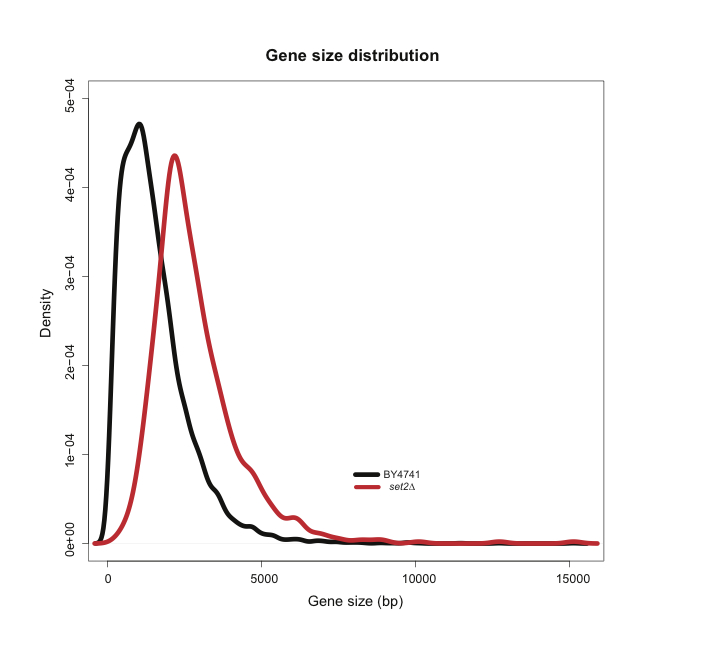

Supplement: Supplementary file 19 [file msb0011-0777-sd19.jpg]

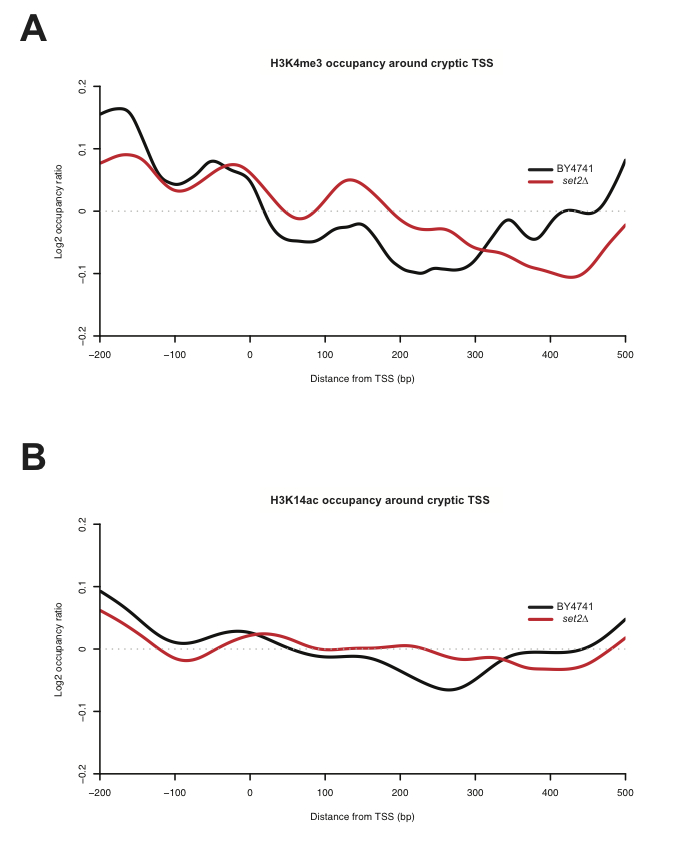

Supplement: Supplementary file 20 [file msb0011-0777-sd20.jpg]

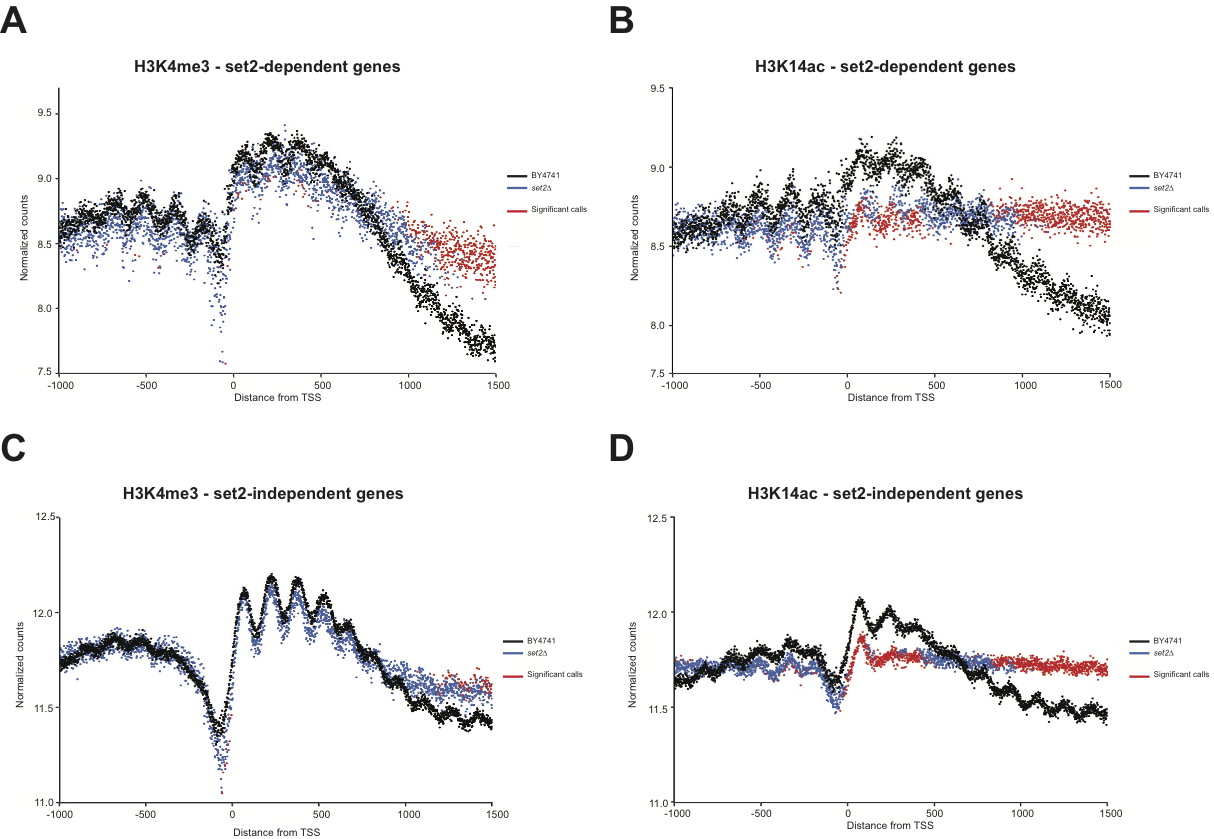

Supplement: Supplementary file 21 [file msb0011-0777-sd21.jpg]

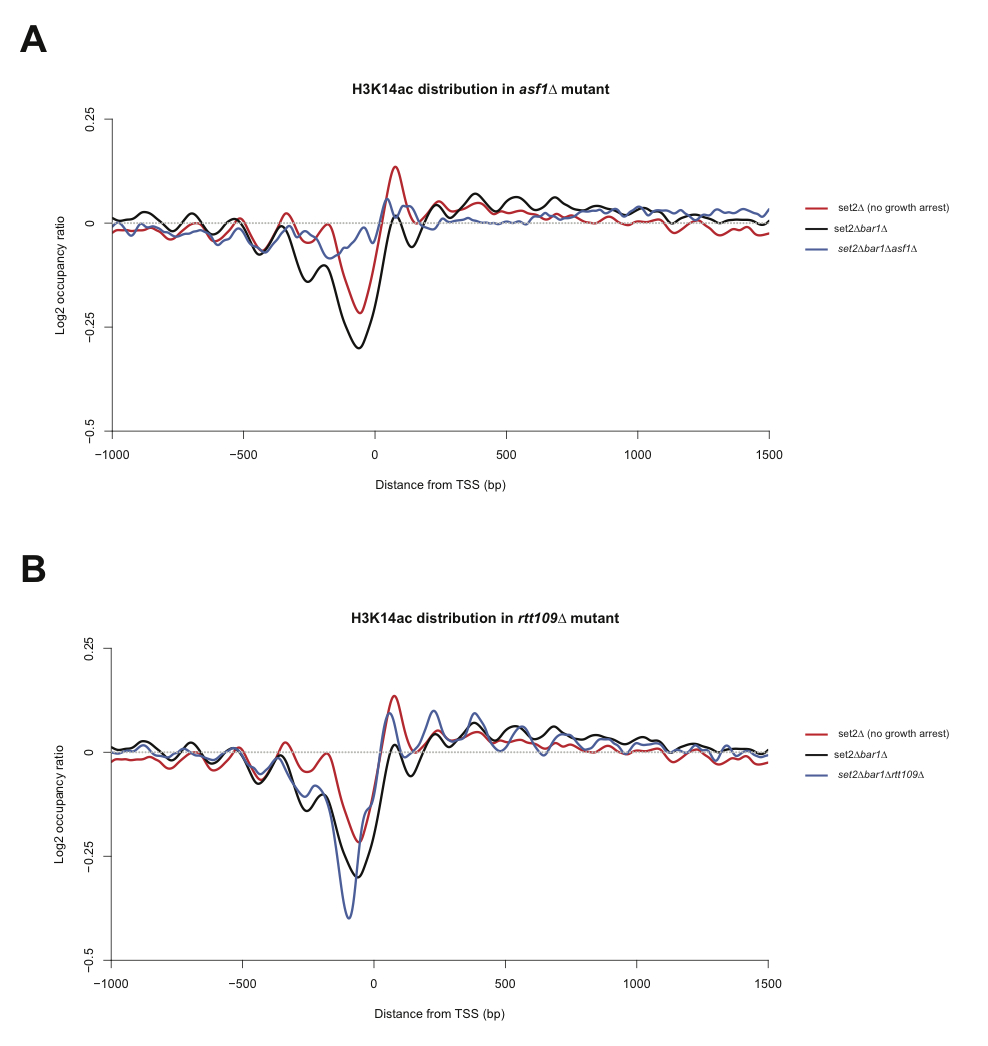

Supplement: Supplementary file 22 [file msb0011-0777-sd22.jpg]

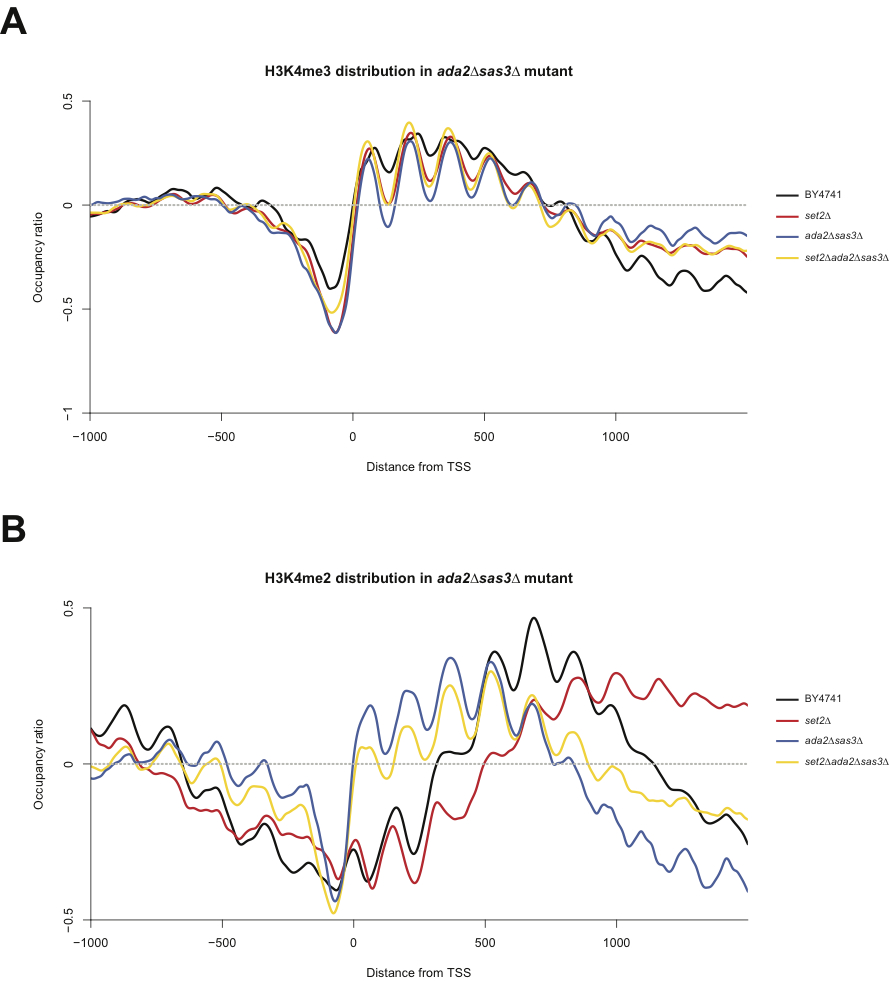

Supplement: Supplementary file 23 [file msb0011-0777-sd23.jpg]

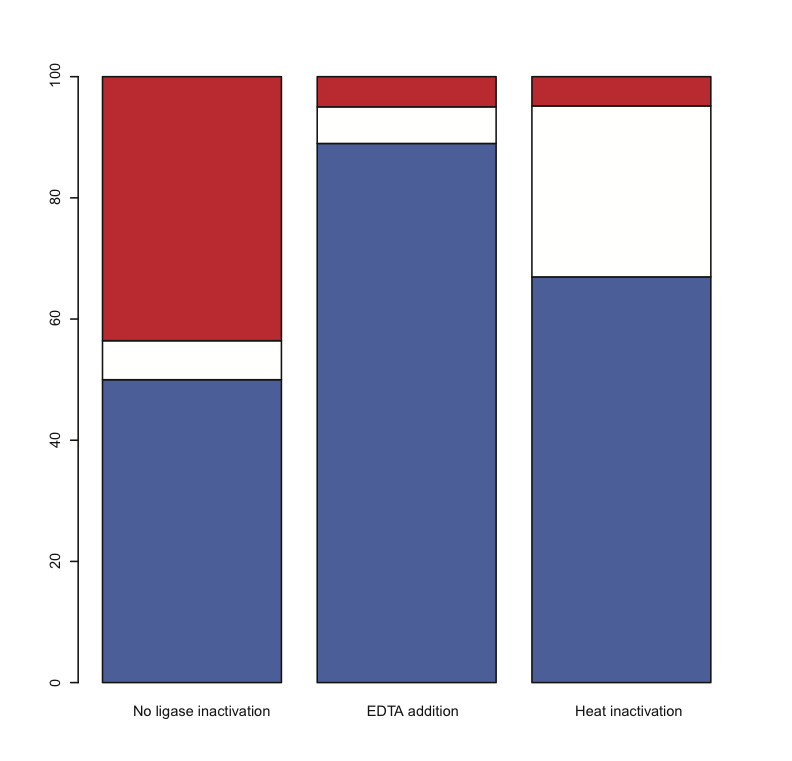

Supplement: Supplementary file 24 [file msb0011-0777-sd24.jpg]

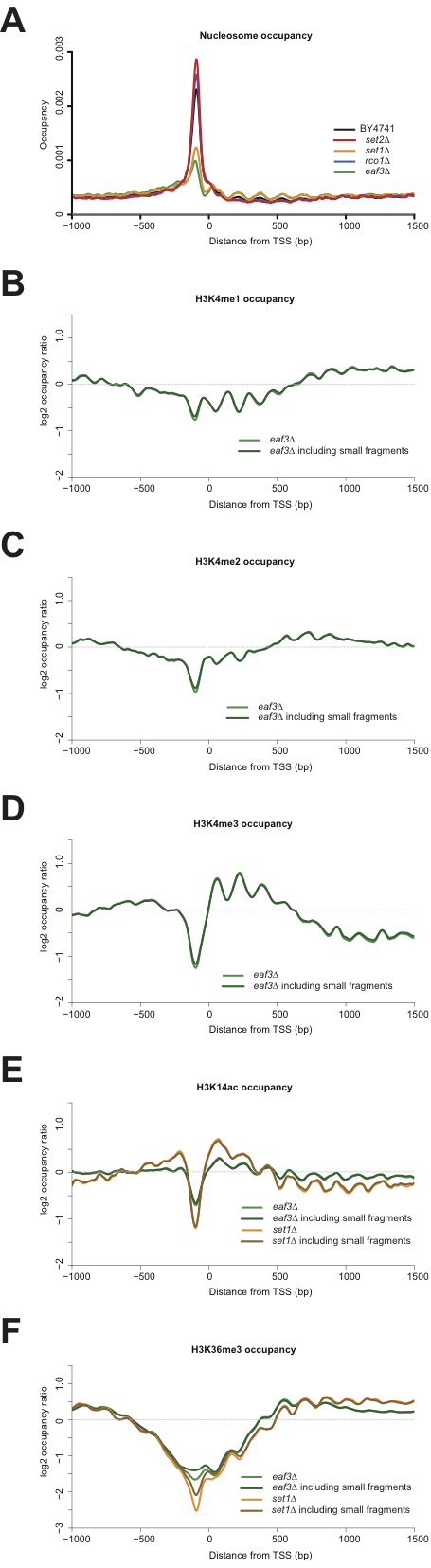

Supplement: Supplementary file 25 [file msb0011-0777-sd25.jpg]
