# Supplementary material for: A high-throughput ChIP-Seq for large-scale chromatin studies
Source: Mol Syst Biol. 2015 Jan 12;11(1):777. doi: 10.15252/msb.20145776 (PMC4332152; doi:10.15252/msb.20145776)
Supplement: Supplementary file 26 [file msb0011-0777-sd26.docx]

Supplementary Legends

Figure S1

(A - L) Size distribution of the nucleosomal fragments obtained with both ChIP-Seq and Bar-ChIP protocols. Fragments for immuno-precipitated and input chromatin are included. Sizes were computed using the genomic coordinates of unambiguously mapped paired-end reads. Vertical red lines represent the cutoff value used to remove DNA fragments not originating from mono-nucleosomes (Material and methods - sequence alignment and read filtering).

Figure S2

Pairwise spearman correlations of the Log2 of reads counts between biological replicates for chromatin inputs obtained using the ChIP (A) and Bar-ChIP (B) protocols as well as for immuno-precipitated chromatin fractions recovered from either ChIP (C) or Bar-ChIP (D) experiments.

Figure S3

(A-F) Scatterplots illustrating the correlation between read counts in the ChIP-Seq and Bar-ChIP datasets. Counts for the input and immuno-precipitated chromatin datasets are included. 100-bp bins were used to compute the counts.

**Figure S4**

Scatterplots illustrating the correlation between H3K4me3 enrichment ratios in the ChIP-Seq and Bar-ChIP datasets. A window of 400-bp (from -5 to +395) around the TSS was used to compute the counts.

Figure S5

Numerical comparison of the ChIP-Seq and Bar-ChIP datasets.

(A-B) Illustration of the normalization of the H3K4me3 counts between two biological replicates using the chromatin input signal for either Bar-ChIP (A) or ChIP-Seq (B) protocols. For each position, the difference between normalized counts of the two replicates (y-axis) was computed and plotted as a function of the mean count between the two values (x-axis). The blue shade illustrates the density of points in a given area of the plot.

1. Dispersion estimation as computed using the DESeq2 package to evaluate the variance-mean relationship in the comparison of Bar-ChIP and ChIP-Seq datasets.
2. Computation of the local FDR from the p-values. Panels show the direct output of the FDR tool. The local FDR used for thresholding is shown at the bottom.

Figure S6

Statistical significance in changes of H3K4me3 distribution between two ChIP-Seq experiments, at a fold change threshold of 0 and 0.05.

1. Normalized H3K4me3 mean counts across biological replicates plotted as a function of the distance to the TSS. Red points represent significant differences between the two conditions. H3K4me3 counts were normalized using the chromatin input counts pondered by the ratio of coverages. Changes in counts were considered significant when the associated local FDR was below 0.2.
2. Normalized H3K4me3 mean counts across biological replicates plotted as a function of the distance to the TSS. Red points represent significant differences between the two conditions. H3K4me3 counts were normalized using the chromatin input counts pondered by the ratio of coverages. Changes in counts were considered significant when the associated local FDR was below 0.2 and when the log2 of the estimated fold change was greater than 0.05.

Figure S7

Percentage of reads attributed to each strain after demultiplexing. After demultiplexing of each sequencing lane, the number of reads attributed to each strain was divided by the total number of reads recovered to correct for potential differences in sequencing depth across lanes. Absolute numbers in million for recovered sequencing reads are indicated.

Figure S8

Distribution of the estimated unique molecules recovered from each sequencing lane. For each dataset, after filtering for pairs of reads stemming from mono-nucleosomes, read pairs defining DNA fragments with more than two identical copies with the same first and last genomic positions were considered as PCR duplicates. Remaining reads were assumed to originate from unique molecules. Their relative abundance in the initial dataset defined the proportion of unique molecules.

**Figure S9**

Bioanalyzer traces for each biological replicate of *set1∆* (A), *eaf3∆* (B) and *set2∆* (C) after MNase digestion.

Figure S10

Nucleosome occupancy around the 5’ nucleosome-depleted region in the 5 yeast strains profiled in the multiplexing experiment.

Figure S11

Size distribution of the nucleosomal fragments obtained from the multiplexed Bar-ChIP experiment. An example of each profiled strain is given. Only fragments from input chromatin are included. Sizes were computed using the genomic coordinates of unambiguously mapped paired-end reads. Red lines represent size thresholds above or below which read pairs were excluded.

**Figure S12**

Statistical significance for changes in H3K4me3 distribution in the wild-type strain profiled by Bar-ChIP in the multiplex experiment (in blue) and in the comparative experiment “Bar-ChIP vs ChIP-Seq" (in black)

**Figure S13**

Residual PTM enrichment patterns for *set1∆* and *set2∆* mutant strains

(A) Residual H3K4me1 enrichment pattern for *set1∆* in comparison with BY4741

(B) Residual H3K4me1 enrichment pattern for *set1∆* in comparison with all other profiled strains

(C) Residual H3K4me2 enrichment pattern for *set1∆* in comparison with BY4741

(D) Residual H3K4me2 enrichment pattern for *set1∆* in comparison with all other profiled strains

(E) Residual H3K4me3 enrichment pattern for *set1∆* in comparison with BY4741

(F) Residual H3K4me3 enrichment pattern for *set1∆* in comparison with all other profiled strains

(G) Residual H3K36me3 enrichment pattern for *set2∆* in comparison with BY4741

(H) Residual H3K36me3 enrichment pattern for *set2∆* in comparison with all other profiled strains

Figure S14

TSS-plots illustrating the distribution of H3K36me3 (A) and H3K4me1 (B) around the TSS, in the wild-type and mutant strains profiled in the multiplexed Bar-ChIP experiment.

**Figure S15**

Gene coverage tracks of 4 chosen loci illustrating the results of the multiplex experiment for H3K4me3. Bedgraph format displaying the number of counts per base pair.

**Figure S16**

TSS-plot representing nucleosome occupancy around the TSS of annotated genes as observed in the chromatin input from the wild-type and *set2∆* mutant strains profiled by classical ChIP-Seq. Occupancy levels are plotted as a function of the distance to the TSS.

Figure S17

Calls for significant changes in H3K14ac (A), H3K4me3 (B) and H3K4me2 (C) distributions between the wild-type strain and the set2Δ mutant. Normalized mean counts across biological replicates were plotted as a function of the distance to the TSS. Red points represent significant differences between the two conditions. Counts were normalized using the chromatin input counts pondered by the ratio of coverages. Changes in counts were considered significant when the associated local FDR was below 0.2 and when the Log2 of the estimated fold change was greater than 0.05.

Figure S18

DESeq2 calls for significant increase in internal transcription initiation sites.

1. Log2 of estimated fold change in counts plotted as a function of the mean counts. Each point represents an annotated feature. Red points represent significant calls for which the fold change is different from 1 and the adjusted p-value is smaller than 0.1.
2. Dispersion estimation as computed using the DESeq2 package to evaluate the variance-mean relationship in the counts datasets for BY4741 and set2Δ.

Figure S19

Gene length distribution for the full Saccharomyces cerevisiae S288c annotated genome (black line) and for the group of Set2-dependent genes (red line).

**Figure S20**

TSS-plots illustrating the distribution of H3K4me3 (A) and H3K14ac (B) around the cryptic TSS, in the wild-type and *set2∆* strains profiled using classical ChIP-Seq.

Figure S21

Calls for significant changes in H3K4me3 (A and C) and H3K14ac (B and D) distributions for the Set2-dependent (A and B) and independent genes (C and D) in the wild-type strain and the set2Δ mutant. Normalized mean counts across biological replicates were plotted as a function of the distance to the TSS. Red points represent significant differences between the two conditions. Counts were normalized using the chromatin input counts pondered by the ratio of coverages. Changes in counts were considered significant when the associated local FDR was below 0.2 and when the log2 of the estimated fold change was greater than 0.05. Set2-dependent and independent genes were called using DESeq2.

Figure S22

TSS-plots illustrating the distribution of H3K14ac in mutants of histone chaperones in a set2Δ background. All occupancy levels were normalized using the chromatin inputs.

The BAR1 gene was deleted in the mutant strains to permit growth arrest by treating yeast cultures with α-factor. set2Δ (no growth arrest) is displayed in red, set2Δbar1Δ is in black while set2Δbar1Δasf1(A) or set2Δbar1Δrtt109(B) are in blue.

Figure S23

TSS-plots illustrating the distribution of H3K4me3 (A) and H3K4me2 (B) in histone acetylation mutant in a set2Δ background. All occupancy levels were normalized using the chromatin inputs. Wild-type background strain BY4741 is displayed in black, set2Δ is in red, the double-mutant ada2Δsas3Δ, which is characterized by a general hypo-acetylation of histone proteins is in blue and the triple mutant set2Δ ada2Δsas3Δ is in yellow.

**Figure S24**

Reads recovered following the sequencing of libraries that were barcoded directly after chromatin fragmentation and pooled together. Proportion of reads with a unique barcode match on each side is indicated in blue, proportion of reads with two different barcodes is indicated in red, and unassigned reads are in white. Three reaction conditions are represented: without ligase inactivation, EDTA mediated inactivation and heat inactivation.

**Figure S25**

(A) Distribution of fragments smaller than 130bp (BY4741, *set2∆*, *rco1∆*) or 100bp (*eaf3∆*, *set1∆*) in the chromatin input for the multiplex experiment.

(B) H3K4me1 occupancies for *eaf3∆* including or not fragments as small as 100bp in the analysis.

(C) H3K4me2 occupancies for *eaf3∆* including or not fragments as small as 100bp in the analysis

(D) H3K4me3 occupancies for *eaf3∆* including or not fragments as small as 100bp in the analysis

(E) H3K14ac occupancies for *eaf3∆* and *set1∆* including or not fragments as small as 100bp in the analysis

(F) H3K36me3 occupancies for *eaf3∆* and *set1∆* including or not fragments as small as 100bp in the analysis

Table S1

Table listing the Set2-dependent genes called in this study.

Table S2

Table providing a description of the yeast strains used in this study.

Table S3

Table listing the antibodies used in this study, together with the providers and catalog numbers.
