# Supplementary material for: A high-throughput ChIP-Seq for large-scale chromatin studies
Source: Mol Syst Biol. 2015 Jan 12;11(1):777. doi: 10.15252/msb.20145776 (PMC4332152; doi:10.15252/msb.20145776)
Supplement: Supplementary file 30 [file msb0011-0777-sd30.pdf]

molecular systems biology

A of high-throughput ChIP-Seq for large-scale chromatin studies

Christophe D. Chabbert, Sophie H. Adjalley, Bernd Klaus, Emilie S. Fritsch, Ishaan Gupta, Vicent Pelechano, Lars M. Steinmetz

Corresponding author: Lars M. Steinmetz, European Molecular Biology Laboratory, Heidelberg

---

Review timeline:

Submission date:	18 September 2014
Editorial Decision:	14 October 2014
Revision received:	31 October 2014
Editorial Decision:	28 November 2014
Revision received:	04 December 2014
Accepted:	05 December 2014

Editor: Thomas Lemberger

Transaction Report:

(Note: With the exception of the correction of typographical or spelling errors that could be a source of ambiguity, letters and reports are not edited. The original formatting of letters and referee reports may not be reflected in this compilation.)

1st Editorial Decision 14 October 2014

Thank you again for submitting your work to Molecular Systems Biology. We have now heard back from the three referees who agreed to evaluate your manuscript. As you will see from the reports below, the referees find the topic of your study of potential interest. They raise, however, important concerns on your work, which should be convincingly addressed in a revision of this work.

Without repeating the comments listed in the reports below, the major issues raised by the reviewers refer to the need to rigorously demonstrate the reliability of the barcoding procedure and exclude artefacts due to the variability of the MNAse digestion step.

On a more editorial leve, we would kindly ask you to include a data availability section at the end of Materials & Methods and list the accession number of the datasets produced in this study. Please include the barcoding information so that the data are interpretable.

If you feel you can satisfactorily deal with these points and those listed by the referees, you may wish to submit a revised version of your manuscript. Please attach a covering letter giving details of the way in which you have handled each of the points raised by the referees. A revised manuscript will be once again subject to review and you probably understand that we can give you no guarantee at this stage that the eventual outcome will be favorable.

© European Molecular Biology Organization

Reviewer #1:

Development of high-throughput ChIP-Seq for large-scale chromatin studies

In this paper, Chabbert et al. describe a method they term Bar-Seq. Bar-Seq is an adaption of a standard MNase-Seq protocol with size-selection that moves the immunoprecipitation step from after MNase digestion to after barcode addition via DNA ligase (library prep is equivalent to an input). By delaying the IP and taking advantage of multiplexing, they can pool dozens of samples and simultaneously IP each of them for the same feature (in this case histone modifications). This pooling drastically decreases the effort and materials involved in producing these ChIP samples. The protocol appears well suited for nucleosome-based samples, as variations in pooling are normalized through a chromatin input control.

Major concerns

1. In Figure 3B, the authors show that for example, H3K4me nucleosomes are depleted in a set1 strain. While that is encouraging, the authors have not demonstrated that the residual nucleosomal pattern in the Bar-ChIP set1 deletion strain (as an example) lacks any resemblance to the H3K4me pattern. That should have been plotted in Figure 4 (normalized to total read count). The same would be true for the others (lack of H3K36me pattern in the set2 strain, etc.). In fact the residual signal in these strains should look like the MNase pattern of samples that were not immunoprecipitated. This is a very important control in that it would demonstrate that the barcodes are true to their original samples. As an example of a contrary possibility, unincorporated adapters might contaminate the samples after pooling, and then possibly get attached to the wrong samples during sample work up. It is not acceptable just to show that there is lower tag recovery in the mutants (Fig. 3B). The system needs to be true for both occupancy (quantitative) and positioning (qualitative).

2. Conceptually I appreciate the protocol optimization, but I am concerned with their sample preps. This study looks at the histone marks of multiple mutant yeast strains to confirm their method. Fig4, SFig8 and SFig9 show different aspects of the nucleosome plots. Fig4A shows a WT nucleosome plot with a very wide -1 nucleosome and practically no NFR; this might be abnormal. The authors comment that their eaf3 and set1 mutants were more sensitive to MNase digestion, however MNase activity can vary somewhat from one experiment to another. So is this sensitivity demonstrated in each biological replicate? The authors should show each individual replicate so that the reader can see the level of variance. Also showing MNase chromatin ladders (gel or bioanalyzer traces) at various MNase concentrations for these different strains would be helpful. The authors use nucleosome counts as the basis for their normalization of histone marks, but it does not appear that they account for the size differences at the -1 nucleosome (i.e. Is a common window size used across samples even those the nucleosome profiles are so different? I don't trust that). There is a concern that the differences that arise from the MNase digestion variability could be greater than the differences they are claiming for changes in histone mark occupancy.

Minor Points

1. After MNase digestion, the MNase is inactivated with 10 mM EGTA. Does this inhibit subsequent enzymatic reactions? If so, how is this alleviated?
2. On Figure 1, include labels of "ChIP-Seq" and "Bar-Seq" after the "Fragmented Chromatin" step for clarity.
3. Figure 2, shouldn't HT-ChIP be labeled "Bar-ChIP"? The term "HT-ChIP" does not appear in the body of the paper.
4. Figure 3B, while seemingly clear, is rather ambiguous. Total counts are considered for each sample, yet it is not clear what proportion of their specific signal is represented in the total counts. The color scale is hard to interpret because it apparently is on a log scale (although it does not say so), and so the fold difference between WT and relevant control mutants is not clear.
5. Sup Figure 10 is missing legends; I assumed they are the same as Figure 4.

Reviewer #2:

This paper presents an interesting and useful variation on ChIP-Seq in which the various chromatin samples are bar-coded before immunoprecipitation. This is a very nice technical advance because it means all the samples can be pooled for immunoprecipitation in the same tube, reducing what is

© European Molecular Biology Organization

probably substantial variation at the IP and subsequent steps. Using this technique, the authors go on to look at deletions of several chromatin-modifying enzymes, revealing some potentially interesting interactions between different histone marks. There is no follow-up to look at the biological effects on gene expression, which would probably be necessary for more general journals, but I don't know MSB and leave it to the editors to decide if that next level of analysis is needed. I have only some minor comments to address:

p6 The text states that H3K4me2 peaks downstream of the TSS has only been shown on single genes, suggesting it has not been previously seen genome-wide. However, the genome-wide studies of Rando (Liu 2005) and Young (Pokholok 2005) have shown this quite clearly.

p9 With no data to support the idea, the discussion about Set1 affecting bidirectional transcription seems to come out of nowhere and is overly speculative. Perhaps the authors have that data somewhere, in which case it should be shown or referenced. If not, these statements should be removed.

Figures 5A and 6A. Something is very confusing here. The K14ac in set2 delta is below the WT curve in 5A, but is above in 6A. If this is not due to mis-labeling, please explain how these two apparently conflicting graphs are used to reach the same conclusion.

Reviewer #3:

In their manuscript entitled "Development of high-throughput ChIP-seq for large-scale chromatin studies', Chabbert et al. present a novel method for ChIP-seq based on introducing a sequencing barcode to MNase-digested chromatin before performing chromatin immune precipitation. This method, named 'Bar-ChIP', allows combining samples with different barcodes during the IP step and therefore reduces the number of parallel reaction in large ChIP-seq studies. Bar-ChIP performs comparable to conventional ChIP-seq protocols. The authors apply their approach to a panel of yeast mutants lacking various components of chromatin modifiers and map a set of five post-translational histone modifications (H3K4me1, me2, me3, H3K4me36, H3K14ac). The authors provide evidence for novel biological insights of the effects of Set2 on histone modifications and its association with the occurrence of cryptic transcripts.
Despite a similar report published recently this method is a valuable addition to the growing number of refined ChIP-seq applications that aim to increase throughput and reproducibility.

Comments

1. In the context of this study the authors pooled all biological replicates into a single IP reaction. This highlights the capacity of their approach to reduce the number of parallel steps and undoubtedly reduced inter-sample variability. However, the authors might want to comment whether such an experimental design is generally desirable, given that it could introduce potential systematic biases that would remain undetectable.

2. Display of data from various genomic backgrounds and histone modifications is almost exclusively based on aggregate plots around TSS. To judge the read distribution on individual loci an example of read distribution displayed in browser tracks would be helpful.

3. Correlation between samples produced by Bar-ChIP and conventional ChIP-seq is high (Fig 2). However, the authors might want to add a correlation based on regions that show some enrichment for H3K4me3 (e.g. Peak calls or TSS regions) to assess the similarity in performance. Genome-wide trends independent of the procedure might contribute substantially to the current correlation measurements.

4. Figure 4a) shows a lower nucleosome density for set1 and eaf3 immediately upstream of the TSS compared to wild type and the other mutants. Compared to Figure 2c these tracks resemble the distribution of the wild type. Are these differences a consequence of variation in the data preparation/analysis or are they a reflection of differences in Bar-ChIP from different pools?

5. Related to Figure 6): the presentation of the analysis of would benefit from adding a plot for

H3K4me3 and H3K14ac density that is centered on 'cryptic TSS' defined based on CAGE tags (i.e. if transcripts emerge from localized sites within a gene and not across broader regions).

Minor comments

1. Figure 2 b): Display of ChIP-seq data in the browser appears fairly noisy. Smoothing might help to improve the visualization (in case none has been applied). Y-axis with the maximum value should be added.

2. The statement on page 7: "Since Bar-ChIP tended to exaggerate both enrichment and depletion signals for histone marks, ..." could be misinterpreted: As a consequence of the normalization enrichment and depletion are not independent of each other (i.e. enrichment in one region will inevitably lead to depletion somewhere else). The authors might want to consider rephrasing.

1st Revision - authors' response 31 October 2014

(see next page)

© European Molecular Biology Organization

RESPONSE TO REVIEWERS

Page and line numbers refer to the file entitled “Revised_MS_Chabbert_et_al_MSB_14_5776”. Text from the initial submission that has now been deleted is indicated in red with a strikethrough, and new text in the revised manuscript is indicated in blue.

Reviewer #1:

In this paper, Chabbert et al. describe a method they term Bar-Seq. Bar-Seq is an adaption of a standard MNase-Seq protocol with size-selection that moves the immunoprecipitation step from after MNase digestion to after barcode addition via DNA ligase (library prep is equivalent to an input). By delaying the IP and taking advantage of multiplexing, they can pool dozens of samples and simultaneously IP each of them for the same feature (in this case histone modifications). This pooling drastically decreases the effort and materials involved in producing these ChIP samples. The protocol appears well suited for nucleosome-based samples, as variations in pooling are normalized through a chromatin input control.

Major concerns

Comment 1. *In Figure 3B, the authors show that for example, H3K4me nucleosomes are depleted in a set1Δ strain. While that is encouraging, the authors have not demonstrated that the residual nucleosomal pattern in the Bar-ChIP set1 deletion strain (as an example) lacks any resemblance to the H3K4me pattern. That should have been plotted in Figure 4 (normalized to total read count). The same would be true for the others (lack of H3K36me pattern in the set2Δ strain, etc.). In fact the residual signal in these strains should look like the MNase pattern of samples that were not immunoprecipitated. This is a very important control in that it would demonstrate that the barcodes are true to their original samples. As an example of a contrary possibility, unincorporated adapters might contaminate the samples after pooling, and then possibly get attached to the wrong samples during sample work up. It is not acceptable just to show that there is lower tag recovery in the mutants (Fig.3B). The system needs to be true for both occupancy (quantitative) and positioning (qualitative).*

Reply: A figure (Supplementary File S13) has now been added to the supplementary section to show the residual nucleosomal pattern obtained for H3K4 methylation in the *set1Δ* strain, as well as for H3K36me3 in *set2Δ*. We show that in either case the nucleosomal pattern, while not resembling that of the corresponding input (i.e. non immuno-precipitated sample), is distinct from that obtained with the wild-type and other mutant strains. The results section of the main manuscript has consequently been extended to address this question and lines 193-196 on page 7 contain the following text: “Additionally, to exclude the possibility of a cross-contamination during sample pooling, the enrichment patterns derived from the remnant reads for H3K4 methylation and H3K36me3 in *set1Δ* and *set2Δ*, respectively, were examined. These did not resemble those of the wild-type strain or of the other mutants (Supplementary File S13)”. Also the supplementary file S15 of a snapshot of coverage tracks for H3K4me3 that is now included as suggested by reviewer #3 (see below) clearly shows a loss of nucleosome pattern for *set1Δ*. This suggests that the residual read counts we obtained for *set1Δ* strain when probing for H3K4 methylation and for *set2Δ* when targeting H3K36me3 do not originate from adapter cross-contamination when pooling the barcoded samples together. We in fact include an additional plot (Supplementary File S24) showing how the inactivation of the DNA ligase after adapter ligation alleviates possible sample contamination with unincorporated adapters as measured by the number of recovered read pairs with two different barcodes. This control experiment consisted in comparing the type of reads (i.e. either reads with a pair of mixed barcodes or reads with identical barcodes on both ends) that were recovered following the sequencing of libraries that were barcoded directly after chromatin fragmentation and pooled together. The barcoding procedure included (or not) a step of DNA ligase inactivation. Results of this control experiment showed that about 45% of the sequencing reads contained mixed barcodes if the DNA ligase was not inactivated after the adapter ligation step, whereas this number dropped to about 2% if the DNA ligase was inactivated. The importance of DNA ligase inactivation is also commented in the revised manuscript in the method section, with the additional sentence on page 11, lines 396-398: “Inactivation of the DNA ligase after the adapter ligation step was particularly important to prevent any further barcoding during the sample pooling and thus any cross-contamination between samples and adapters (Supplementary File S24)”.

Additionally, we propose below a numerical estimation of the sequencing reads with misincorporated DNA barcodes, which is based on the number of read pairs we recovered with two different barcodes. If the 15 barcodes we used in the study were to be randomly ligated to the wrong DNA sample fragments, we would recover sequencing reads with $\binom{15}{2} + \binom{15}{1} = 120$ possible barcode combinations, 15 of which would not be detectable, as they would correspond to the ligation of the same barcode to both ends of one fragment. Therefore, by counting read pairs with two different barcodes,

we are sampling $\frac{120-15}{120} * 100 = 87.5\%$ of the fragments that are not properly barcoded, which corresponds to an average of 3.5 million reads in each of the sequencing lanes for immunoprecipitated samples. We can therefore estimate that $\frac{(1-0.875)\times 3.5}{0.875} = 0.50$ million reads approximately may potentially be attributed to the wrong sample. Assuming that these reads are evenly distributed across all 15 samples, that would represent ~33 000 reads per sample, which is less than the residual fragments recovered in the *set1Δ* and *set2Δ* strain (average numbers X and Y, respectively). As a conclusion, our numerical estimations coupled with the aforementioned experiment assessing the impact of ligase inactivation strongly suggest that the reads recovered in the *set1Δ* and *set2Δ* mutants do not originate from random ligation of barcodes but are rather true residual products recovered after immunoprecipitation.

Comment 2. *Conceptually I appreciate the protocol optimization, but I am concerned with their sample preps. This study looks at the histone marks of multiple mutant yeast strains to confirm their method. Fig4, SFig8 and SFig9 show different aspects of the nucleosome plots. Fig4A shows a WT nucleosome plot with a very wide -1 nucleosome and practically no NFR; this might be abnormal. The authors comment that their eaf3Δ and set1Δ mutants were more sensitive to MNase digestion, however MNase activity can vary somewhat from one experiment to another. So is this sensitivity demonstrated in each biological replicate? The authors should show each individual replicate so that the reader can see the level of variance. Also showing MNase chromatin ladders (gel or bioanalyzer traces) at various MNase concentrations for these different strains would be helpful. The authors use nucleosome counts as the basis for their normalization of histone marks, but it does not appear that they account for the size differences at the -1 nucleosome (i.e. Is a common window size used across samples even those the nucleosome profiles are so different? I don't trust that). There is a concern that the differences that arise from the MNase digestion variability could be greater than the differences they are claiming for changes in histone mark occupancy.*

Reply: We have now added to the supplementary section a file corresponding to the bioanalyzer traces obtained for each biological replicate of *set1Δ* and *eaf3Δ* mutant strains (Supplementary File S9), which we refer to on page 6, line 174 of the main manuscript. As a comparison, we are also adding the profiles obtained for *set2Δ* biological replicates, to which those of BY4741 and *rco1Δ* are similar. These profiles clearly show that parallel enzymatic treatment of all samples with identical MNase concentration and treatment duration led to greater digestion of *set1Δ* and *eaf3Δ* chromatin. This suggests increased sensitivity to the enzyme for these 2 mutants, which is reproducible as indicated by the great similarity of the chromatin ladder profiles between all three biological replicates. Again, we would like to highlight the fact that all samples for the multiplexing experiment were treated together with the same batch of MNase, for the same duration, using the same enzyme concentration.
We agree with Reviewer #1 that the ideal methodology would be to systematically establish the adequate conditions of MNase treatment for each strain using various enzyme concentrations. However, the purpose of our experimental approach is to provide a fast way to profile histone marks in several strains at once. We show that enzymatic treatment using standard conditions (as established with chromatin from the wild-type strain) does not impair our capacity to properly capture profiles of histone marks. These profiles were later confirmed for the wild-type and *set2Δ* strains using a standard ChIP-Seq approach. We kindly draw the reviewers' attention to the profiles of nucleosome occupancy derived from this follow-up experiment that have now been included as Supplementary File S16 (referred to on page 7, line 222) and show the typical -1 nucleosome in both strains. We therefore consider that, although wild-type, *rco1Δ* and *set2Δ* mutant strains harbored an atypically wide -1 nucleosome in the multiplex experiment as shown on Figure 4A (N.B.: the NFR is clearly visible in Supplementary File S10), we were able to properly profile all selected histone marks in the strains. We also would like to politely insist on the fact that our observations focus on the TSS, +1 and downstream nucleosomes, and that the typical and expected enrichment patterns for all histone marks profiled in our study were observed in the wild-type strain, whether it harbored a typical -1 nucleosome profile or not. Nevertheless, we have now addressed this matter in the revised manuscript as follows and have included an additional supplementary figure (Supplementary File S12). On page 6, lines 176-178, the sentence now reads: "In contrast, each other mutant exhibited typical nucleosome organization around the TSS (Figure 4A and Supplementary File S10), except for an unusually wider -1 nucleosome". On page 7, lines 190-192 contain the following additional text: "Despite the aforementioned wider -1 nucleosome, comparison of the H3K4me3 enrichment patterns obtained in both comparative and multiplex experiments did not show any significant difference (Supplementary File S12)".
Finally, a different window size was in fact used for *eaf3Δ* and *set1Δ* inputs to take in consideration the difference in nucleosome patterns between the two mutants and the other strains. Supplementary Figure S11 has been corrected as it originally suggested that the same window had been used for all profiled strains. We have also added the following sentences in the method section on page 13, lines 479-483 to further detail the approach used in our data analysis: "To

take into consideration the variations in MNase sensitivity between the profiled yeast strains and therefore the difference in the size of the chromatin fragments, notably between *set1Δ* and *eaf3Δ* mutants and all other strains (Supplementary File S11), an extended window size for mono-nucleosome fragments was used to generate nucleosome counts for the inputs (to include fragments as small as 100bp)". Furthermore, we have included an additional figure in the supplementary section showing that a shift of the window size used to define mono-nucleosome does not change our computation of the histone occupancy profiles and have added the following sentence in the method section on page 13, lines 483-484: "Use of the same window size for the IP fractions did not however modify the computation of histone occupancies (Supplementary File S25)"

Minor Points

Comment 1. *After MNase digestion, the MNase is inactivated with 10 mM EGTA. Does this inhibit subsequent enzymatic reactions? If so, how is this alleviated?*
Reply: We did not observe any inhibition of the subsequent enzymatic reactions after the addition of 10mM EGTA to inactivate the MNase, as indicated by the proper amplification of input libraries right after adapter ligation. In fact, we perform the MNase digestion in a buffer with high concentrations of Ca^{2+} and Mg^{2+} ions. With a much greater affinity for Ca^{2+} than for Mg^{2+}, EGTA does not appear to chelate the Mg^{2+} ions that are additionally present in the reaction buffers used for the subsequent enzymatic reactions. The following sentence has been added in the methods section on page 11, lines 387-389 to address this topic: "Note that given the stronger affinity of EGTA for Ca^{2+} than Mg^{2+} ions, this concentration is sufficient to inactivate the MNase without inhibiting any of the downstream enzymatic reactions".

Comment 2. *On Figure 1, include labels of "ChIP-Seq" and "Bar-Seq" after the "Fragmented Chromatin" step for clarity.*
Reply: Figure 1 has now been modified to include the labels at a clearer position.

Comment 3. *Figure 2, shouldn't HT-ChIP be labeled "Bar-ChIP"? The term "HT-ChIP" does not appear in the body of the paper.*
Reply: This typo has now been corrected.

Comment 4. *Figure 3B, while seemingly clear, is rather ambiguous. Total counts are considered for each sample, yet it is not clear what proportion of their specific signal is represented in the total counts. The color scale is hard to interpret because it apparently is on a log scale (although it does not say so), and so the fold difference between WT and relevant control mutants is not clear.*
Reply: We have added the following comment in the legend for Figure 3B (page 20, lines 745-749) to clarify the color scale: "Note that each *set1Δ* library represents less than 0.17% of the total number of reads recovered for the IPs against H3K4 methylation while the *set2Δ* libraries represent no more than 0.8% of the total reads recovered for the IP against H3K36me3. Absolute numbers for recovered sequencing reads are indicated in Supplementary File S7". We would like to kindly remind the reviewer that absolute values for each sample should only be considered in light of the total read counts in each sequencing lane and analyzed as a fraction of the total number of reads recovered as indicated by the color scale.

Comment 5. *Sup Figure 10 is missing legends; I assumed they are the same as Figure 4.*
Reply: Legends have now been added to Supplementary File S10 (now Supplementary File S14).

Reviewer #2:

This paper presents an interesting and useful variation on ChIP-Seq in which the various chromatin samples are bar-coded before immunoprecipitation. This is a very nice technical advance because it means all the samples can be pooled for immunoprecipitation in the same tube, reducing what is probably substantial variation at the IP and subsequent steps. Using this technique, the authors go on to look at deletions of several chromatin-modifying enzymes, revealing some potentially interesting interactions between different histone marks. There is no follow-up to look at the biological effects on gene expression, which would probably be necessary for more general journals, but I don't know MSB and leave it to the editors to decide if that next level of analysis is needed. I have only some minor comments to address:

Comment 1. *p6 The text states that H3K4me2 peaks downstream of the TSS has only been shown on single genes,*

suggesting it has not been previously seen genome-wide. However, the genome-wide studies of Rando (Liu 2005) and Young (Pokholok 2005) have shown this quite clearly.
Reply: We have modified the text for lines 185-188 (pages 6-7), which now reads: "High levels of H3K4me2 were located about 500-bp after annotated transcription initiation sites, as previously reported in single gene and genome-wide studies (Liu et al., 2005; Ng et al., 2003; Pokholok et al., 2005; Santos-Rosa et al., 2002)"

Comment 2. *p9 With no data to support the idea, the discussion about Set1 affecting bidirectional transcription seems to come out of nowhere and is overly speculative. Perhaps the authors have that data somewhere, in which case it should be shown or referenced. If not, these statements should be removed.*
Reply: Given that we do not have at the moment any additional data that would support the proposed hypothesis that bidirectional promoters are affected in *set1Δ*, we agree to remove the section. The text for lines 326-331 on page 9 - 10 now reads: "In fact, both histone marks appeared to be enriched at the -1 nucleosome in comparison to the wild-type strain, which could represent disequilibrium in bidirectional promoter activity as previously observed in the context of histone hyperacetylation (Schuettengruber, 2002). ~~Set1p-mediated H3K4 dimethylation was reported to recruit the Set3p histone deacetylase to promoter-proximal regions (Kim and Buratowski, 2009). It is possible that Set3p also gets recruited to bidirectional promoters to maintain polarization of transcription under certain conditions. Most bidirectional promoters regulate the expression of concurrently activated genes (Lin et al., 2007). However, previous studies have reported that in the context of bidirectional promoter sharing, histone hyperacetylation may favor transcription in one direction (Schuettengruber, 2002)~~. Further investigation will be needed to assess whether ~~a similar mechanism~~ such a mechanism plays a role in the localized PTM enrichment we observed in *set1Δ*."

Comment 3. *Figures 5A and 6A. Something is very confusing here. The K14ac in set2 delta is below the WT curve in 5A, but is above in 6A. If this is not due to mis-labeling, please explain how these two apparently conflicting graphs are used to reach the same conclusion.*
Reply: Figure 5A represents all genes, thus include both Set2-dependent and Set2-independent genes. What figure 6A shows is that the signals observed on Figure 5A are mainly driven by Set2-dependent genes (same color code as in Figure 5A, with the wild-type strain in black and the *set2Δ* strain in red), although a small difference in the pattern of H3K14 acetylation is already observed between the two strains (wild-type strain in blue and *set2Δ* mutant in yellow) for Set2-independent genes. We have modified the title of Figure 6 for lines 774-777 on page 20, which now reads: "TSS-plots representing the distribution of H3K14ac and H3K4me3 ~~in different group of genes in the wild-type strain and set2Δ mutant~~ for genes grouped as either Set2-dependent or Set2-independent in the wild-type strain and *set2Δ* mutant.
We have also added the following description of the figure to avoid any confusion: "Set2-dependent genes are either in black (for BY4741) or red (for *set2Δ*), while Set2-independent genes are represented as a blue (for BY4741) or yellow line (for *set2Δ*)".

Reviewer #3:

In their manuscript entitled "Development of high-throughput ChIP-seq for large-scale chromatin studies', Chabbert et al. present a novel method for ChIP-seq based on introducing a sequencing barcode to MNase-digested chromatin before performing chromatin immune precipitation. This method, named 'Bar-ChIP', allows combining samples with different barcodes during the IP step and therefore reduces the number of parallel reaction in large ChIP-seq studies. Bar-ChIP performs comparable to conventional ChIP-seq protocols. The authors apply their approach to a panel of yeast mutants lacking various components of chromatin modifiers and map a set of five post-translational histone modifications (H3K4me1, me2, me3, H3K4me36, H3K14ac). The authors provide evidence for novel biological insights of the effects of Set2 on histone modifications and its association with the occurrence of cryptic transcripts.
Despite a similar report published recently this method is a valuable addition to the growing number of refined ChIP-seq applications that aim to increase throughput and reproducibility.

Comments

Comment 1. *In the context of this study the authors pooled all biological replicates into a single IP reaction. This highlights the capacity of their approach to reduce the number of parallel steps and undoubtedly reduced inter-sample variability. However, the authors might want to comment whether such an experimental design is generally desirable, given that it could introduce potential systematic biases that would remain undetectable.*

Reply: We have added a sentence in the discussion section to reemphasize the necessity of confirming the trends observed with Bar-ChIP, which is a powerful approach to obtain genome-wide patterns of histone modifications albeit at a modest resolution and with potential biases, notably inherent to the barcoding step. Lines 305-310 on page 9 include now the following sentence: "The limited number of estimated unique molecules retrieved per IP, reflect a moderate complexity of the sequencing libraries. This might originate from a limited efficiency of the nucleosome barcoding step, added to a possible preference of the ligase for certain populations of nucleosomes. Given the aforementioned potential biases and the modest resolution of the data, Bar-ChIP is an ideal approach to obtain a quick genome-wide overview of histone PTM enrichment patterns. Interesting trends may then be confirmed by complementary experiments".

Comment 2. *Display of data from various genomic backgrounds and histone modifications is almost exclusively based on aggregate plots around TSS. To judge the read distribution on individual loci an example of read distribution displayed in browser tracks would be helpful*.
Reply: We have now added a supplementary figure (Supplementary File S15) showing several gene tracks as an illustration of read distributions obtained with the Bar-ChIP approach when comparing H3K4me3 between the 5 profiled yeast strains. The manuscript has also been modified on page 7 lines 206-210 to refer the readers to this additional figure: "While the main peak of H3K4me3 occupancy was present near the 5' end of genes, similarly to the wild-type profile, a modest enrichment of H3K4 trimethylation was detected at the 3' end of genes, beyond the first 500-bp. This was also confirmed by manual examination of gene coverage tracks (Supplementary File S15)".

Comment 3. *Correlation between samples produced by Bar-ChIP and conventional ChIP-seq is high (Fig 2). However, the authors might want to add a correlation based on regions that show some enrichment for H3K4me3 (e.g. Peak calls or TSS regions) to assess the similarity in performance. Genome-wide trends independent of the procedure might contribute substantially to the current correlation measurements.*
Reply: We have now added a supplementary figure (Supplementary File S4) showing the correlation between the two methods calculated using regions of H3K4me3 enrichment around the TSS. The correlation coefficient values remain high, supporting our statement that Bar-ChIP and conventional ChIP-Seq are similarly efficient. We have also modified the sentence on page 4, lines 77-79 in the revised manuscript as follows: "Signals for the presence of the H3K4me3 mark were equally well recovered by the two methods as confirmed by the high correlation obtained for regions with the PTM enrichment (Supplementary File S4) and by manual inspection of the coverage tracks for selected loci"

Comment 4. *Figure 4a) shows a lower nucleosome density for set1Δ and eaf3Δ immediately upstream of the TSS compared to wild type and the other mutants. Compared to Figure 2c these tracks resemble the distribution of the wild type. Are these differences a consequence of variation in the data preparation/analysis or are they a reflection of differences in Bar-ChIP from different pools?*
Reply: As discussed above in our response to the Major comment 2 from reviewer #1, we believe that the trends observed in the multiplex experiment stem from variations in the sample preparation. However, as depicted earlier, we do not think that these differences impair our capacity to capture histone PTM trends genome-wide, nor the conclusions that may be drawn from the analysis.

Comment 5. *Related to Figure 6): the presentation of the analysis of would benefit from adding a plot for H3K4me3 and H3K14ac density that is centered on 'cryptic TSS' defined based on CAGE tags (i.e. if transcripts emerge from localized sites within a gene and not across broader regions)*.
Reply: We have now added a figure in the Supplementary section (Supplementary File S20) that shows the patterns of H3K4me3 and H3K14ac around the TSS of the cryptic transcripts that emerge in the *set2Δ* mutant. We are referring the readers to this figure in the main text on page 8, line 242-243: "Occupancy profiles of H3K14ac and H3K4me3 were then computed for this group of genes in both wild type and mutant strains (Figure 6, Supplementary Files S20 and S21)".

Minor comments

Comment 1. *Figure 2 b): Display of ChIP-seq data in the browser appears fairly noisy. Smoothing might help to improve*

the visualization (in case none has been applied). Y-axis with the maximum value should be added.
Reply: We have now added the maximum RPKM values on the Y-axis for Figure 2B. We do not feel however that smoothing of the data is required in the present case given the high coverage we have obtained for the whole genome.

Comment 2. *The statement on page 7: "Since Bar-ChIP tended to exaggerate both enrichment and depletion signals for histone marks, ..." could be misinterpreted: As a consequence of the normalization enrichment and depletion are not independent of each other (i.e. enrichment in one region will inevitably lead to depletion somewhere else). The authors might want to consider rephrasing.*
Reply: Reviewer #3 is correct and we have rephrased the text on page 7, lines 219-220 to avoid any confusion, such that it now reads: "Since ~~Bar-ChIP tended to exaggerate both enrichment and depletion signals for histone marks~~ signals for histone marks enrichment and consequently depletion tend to be exaggerated with Bar-ChIP…"

2nd Editorial Decision 28 November 2014

Thank you again for submitting your work to Molecular Systems Biology. We have now heard back from the two referees who accepted to evaluate the study. As you will see, the referees are now rather supportive. Reviewer #2 still raises a few issues that we would ask you to address in a last round of minor revision of the study. The comments refer to the need of further clarifications.

We would also suggest to modify slightly the title of your study: "A high-throughput ChIP-Seq method for large-scale chromatin studies".

Thank you for submitting this paper to Molecular Systems Biology.

Reviewer #1:

Specific comments Revision 2:
1. How long was each heat inactivation? Wouldn't that cause reversal of the formaldehyde crosslinking?

2. Sentence starting with "To ensure consistency" needs "were".

3. Not clear why Fig. S9 does not report the bioanalyzer trace for the other strains. The standard here is BY4741, and yet its trace is not shown. This is important because comparison to the Set2 traces might suggest that the comparison state (BY4741) is under-digested with Mnase. While this issue has nothing to do with bar-chip per se, it does give the impression that there is no NFR in WT, and in the rco1 and set2 mutants, despite the authors description of them as less pronounced. While this is true in a relative sense, one comes away with the impression that Rco1 and Set2 are important for creating NFRs (not a conclusion drawn by the authors).

4. In Fig. S10, I don't see how this can be interpretted as "unusually wider -1 nucleosome", as opposed to a more heterogeneous distribution in a gene-average analysis. What is the interpretation of a wide nucleosome? Isn't it essentially a consequence of how much Mnase is used, and thus a technical issue rather than a biological issue?

5. This issue regarding potential cross contamination by examining the distribution of histone marks is not convincing one way or the other for Fig. S13A-D because the patterns are not all that different from each other and not that intrinsically unique. If one looks at Figure S13E,F, the loss of set1 still shows nucleosomal enrichment at the 5' end of genes that looks very similar to BY4741. Figure S10G,H looks more convincing. Thus, the results are mixed: potential cross-contamination in some cases and less in others. Much of this could be due to the relative numbers of tags recovered, since Fig. S7 shows that set2 deletion strain has relatively high number of recovered reads and set1 deletion strains has very low reads (and thus more susceptible to contamination). Thus one could come to a very incorrect conclusion regarding the requirement for Set1 in H3K4me3, unless they pay attention to the total number of tags recovered. So, I'm not saying that bar-chip does not work, its just that one needs to be very careful about situations where there is low read recovery.

6. In Fig. 4D,E, the difference between H3K4me2,3 patterns in WT vs set2 deletion, is very small. It is not clear whether this is meaningful or not. The authors simply state that there is a difference without really making a conclusion.

7. On what basis is the use of the word "exaggerated" based: "Since signals for histone marks enrichment and consequently depletion tend to be exaggerated with Bar-ChIP, ..." Also I don't think Figure S16 matches with the call-out in the text. (Note that throughout the manuscript none of the figures or their file names indicate what figure number they are. So, I had to inferred this from the file order, which may be more linked to the upload order than actual figure #).

Reviewer #3:

The authors adequately addressed my comments.

2nd Revision - authors' response 04 December 2014

We were delighted to learn that the reviewers were supportive of our study and we are pleased to submit a revised version of the manuscript to address the minor issues raised by one of the referees. You will find below the amendments we made to the manuscript and our response to each of the referee's comments. Following the same nomenclature as before, the text that has been deleted in the revised manuscript is indicated in red with a strikethrough, and new text is in blue. In agreement with the authors' guidelines, all "Supplementary File Sxx" have been renamed "Figure Exx". Also, following the editorial recommendation, the title of the manuscript has been changed as indicated above.

To address **comment 1**: "*How long was each heat inactivation? Wouldn't that cause reversal of the formaldehyde crosslinking?"* we have added a sentence in the Material and methods section specifying the duration of the ligase heat inactivation: "Inactivation of the DNA ligase by incubating samples at 65oC for 15 minutes after the adapter ligation step was particularly important to prevent any further barcoding during the sample pooling and thus any cross-contamination between samples and adapters (Figure E24)." (lines 398-401, page 11).

We do not think that this condition permits a reversal of the formaldehyde crosslinking and have added a sentence in the text to raise this matter: "Note that the length of incubation was not sufficient to promote reverse crosslinking of the samples, usually obtained after 12-14hrs." (lines 401-402, page 11). On a side note, we personally have noticed that even after two hours, chromatin crosslinking was not reversed.

We also thank the reviewer for identifying the typo as in **comment 2**: "*Sentence starting with "To ensure consistency" needs "were""*. This has now been corrected in line 115, page 5.

The referee raised the following point in **comment 3**: "*Not clear why Fig. S9 does not report the bioanalyzer trace for the other strains. The*

Heidelberg, December 4th 2014,

standard here is BY4741, and yet its trace is not shown. This is important because comparison to the Set2 traces might suggest that the comparison state (BY4741) is under-digested with Mnase. While this issue has nothing to do with bar-chip per se, it does give the impression that there is no NFR in WT, and in the rco1 and set2 mutants, despite the authors description of them as less pronounced. While this is true in a relative sense, one comes away with the impression that Rco1 and Set2 are important for creating NFRs (not a conclusion drawn by the authors)". We found this concern legitimate and have now included all bioanalyzer traces in Figure E9.

Given the reviewer's previous comment (comment #2 in the previous round of revision) "Fig4A shows a WT nucleosome plot with a very wide -1 nucleosome and practically no NFR", we had accordingly updated the manuscript to address this matter. However, we have modified the text to avoid any confusion in the interpretation of our observations, which is a concern that was raised by the referee in **comment 4**: "*In Fig. S10, I don't see how this can be interpreted as "unusually wider -1 nucleosome", as opposed to a more heterogeneous distribution in a gene-average analysis. What is the interpretation of a wide nucleosome? Isn't it essentially a consequence of how much Mnase is used, and thus a technical issue rather than a biological issue?*". We have now described this particularity as "an unusually wider profile of the -1 nucleosome" that we indeed attribute to technical variations; the main text has been modified to now include the following sentences: "We attribute this difference to a greater heterogeneity of the fragments obtained by MNase digestion of the corresponding regions." (lines 176-178, page 6) and "Despite the aforementioned wider profile of the -1 nucleosome, comparison of the H3K4me3 enrichment patterns obtained in both

comparative and multiplex experiments did not show any significant difference (Figure E12)" on lines 190-192, page 7.

We think that **comment 5** raises a valid but sensitive point: "*This issue regarding potential cross contamination by examining the distribution of histone marks is not convincing one way or the other for Fig. S13A-D because the patterns are not all that different from each other and not that intrinsically unique. If one looks at Figure S13E,F, the loss of set1 still shows nucleosomal enrichment at the 5' end of genes that looks very similar to BY4741. Figure S10G,H looks more convincing. Thus, the results are mixed: potential cross-contamination in some cases and less in others. Much of this could be due to the relative numbers of tags recovered, since Fig. S7 shows that set2 deletion strain has relatively high number of recovered reads and set1 deletion strains has very low reads (and thus more susceptible to contamination). Thus one could come to a very incorrect conclusion regarding the requirement for Set1 in H3K4me3, unless they pay attention to the total number of tags recovered. So, I'm not saying that bar-chip does not work, its just that one needs to be very careful about situations where there is low read recovery*".

Indeed, we believe that our data do not allow distinguishing between possible cross-contamination, antibody cross-reaction or real signal. Nevertheless, we have modified the main text and added a sentence to elaborate on the matter and draw the reader's attention to the importance of taking into account the sequencing coverage: "Additionally, to exclude the possibility of assess the possibility of cross-contamination during sample pooling, the enrichment patterns derived from the remnant reads for H3K4 methylation and H3K36me3 in *set1*Δ and *set2*Δ, respectively, were examined. These did not resemble those of the wild-type strain or of the other mutants (Supplementary File S13). These generally did not resemble those of the wild-

type strain or of the other mutants (Figure E13), although traces of H3K4 methylation were still detected in *set1*Δ (Figure E13E-F), albeit corresponding to a very low number of sequencing reads (Figure E7)." (lines 193-197, page 7)

To address **comment 6**: "*In Fig. 4D,E, the difference between H3K4me2,3 patterns in WT vs set2 deletion, is very small. It is not clear whether this is meaningful or not. The authors simply state that there is a difference without really making a conclusion",* we have now extended our statement to give a clear conclusion on our observations in the multiplexing experiment: "In the *set2*Δ mutant, distributions of H3K4me3, H3K4me2, and H3K4me1 to a lesser extent, differed from those observed in the wild-type strain (Figures 4D-E, Figure E14), suggesting that the deletion of *SET2* impacts the methylation profile of H3K4." (lines 206-208, page 7).

We have also modified the text to be more specific regarding the significance of the observed differences: "Similarly to what we observed with Bar-ChIP, both H3K4me3 and H3K4me2 marks were significantly enriched near the 3' end of annotated genes in the set2Δ mutant and these differences were statistically significant (Figures 5B-C and Figure E17-B and -C)" (lines 226-229, page 7).

Finally to answer the referee's **comment 7**: "*On what basis is the use of the word "exaggerated" based: "Since signals for histone marks enrichment and consequently depletion tend to be exaggerated with Bar- ChIP, ...",* we have modified the sentence as follows to clarify the statement: "Since signals for histone marks enrichment and consequently depletion tend to be exaggerated with Bar-ChIP as shown above in the experiment comparing our method to classical ChIP-Seq, distributions of H3K4me3, H3K4me2, and H3K4ac marks were independently corroborated for the wild- type and set2Δ strains with the latter approach" (lines 220-223, page 7). We have also added some text to better refer to Figure E16 following the reviewer's comment "*Also I don't think Figure S16 matches with the call-out in the text. (Note that throughout the manuscript none of the figures or their file names indicate what figure number they are. So, I had to inferred this from the file order, which may be more linked to the upload order than actual figure #*)". The text now reads: "No difference in nucleosome occupancy was detected between the two strains (Figure E16)." (lines 223-224, page 7).

We hope that the revised version of the manuscript and our responses to the reviewer will satisfy the requests.
